# Activity-State Dependent Reversal of Ketamine-Induced Resting State EEG Effects by Clozapine and Naltrexone in the Freely Moving Rat

**DOI:** 10.3389/fpsyt.2022.737295

**Published:** 2022-01-27

**Authors:** Christien Bowman, Ulrike Richter, Christopher R. Jones, Claus Agerskov, Kjartan Frisch Herrik

**Affiliations:** ^1^Faculty of Psychology and Neuroscience, Maastricht University, Maastricht, Netherlands; ^2^Bio Imaging Laboratory, Faculty of Pharmaceutical, Biomedical and Veterinary Sciences, University of Antwerp, Antwerp, Belgium; ^3^Department of Circuit Biology, Lundbeck, Copenhagen, Denmark; ^4^Department of Pharmacokinetic and Pharmacodynamic Modeling and Simulation, Lundbeck, Copenhagen, Denmark

**Keywords:** NMDAR (NMDA receptor), resting state EEG, translational biomarker, schizophrenia, antidepressant, naltrexone, clozapine, ketamine

## Abstract

Ketamine is a non-competitive N-Methyl-D-aspartate receptor (NMDAR) antagonist used in the clinic to initiate and maintain anaesthesia; it induces dissociative states and has emerged as a breakthrough therapy for major depressive disorder. Using local field potential recordings in freely moving rats, we studied resting state EEG profiles induced by co-administering ketamine with either: clozapine, a highly efficacious antipsychotic; or naltrexone, an opioid receptor antagonist reported to block the acute antidepressant effects of ketamine. As human electroencephalography (EEG) is predominantly recorded in a passive state, head-mounted accelerometers were used with rats to determine active and passive states at a high temporal resolution to offer the highest translatability. In general, pharmacological effects for the three drugs were more pronounced in (or restricted to) the passive state. Specifically, during inactive periods clozapine induced increases in delta (0.1–4 Hz), gamma (30–60 Hz) and higher frequencies (>100 Hz). Importantly, it reversed the ketamine-induced reduction in low beta power (10–20 Hz) and potentiated ketamine-induced increases in gamma and high frequency oscillations (130–160 Hz). Naltrexone inhibited frequencies above 50 Hz and significantly reduced the ketamine-induced increase in high frequency oscillations. However, some frequency band changes, such as clozapine-induced decreases in delta power, were only seen in locomoting rats. These results emphasise the potential in differentiating between activity states to capture drug effects and translate to human resting state EEG. Furthermore, the differential reversal of ketamine-induced EEG effects by clozapine and naltrexone may have implications for the understanding of psychotomimetic as well as rapid antidepressant effects of ketamine.

## Graphical Abstract

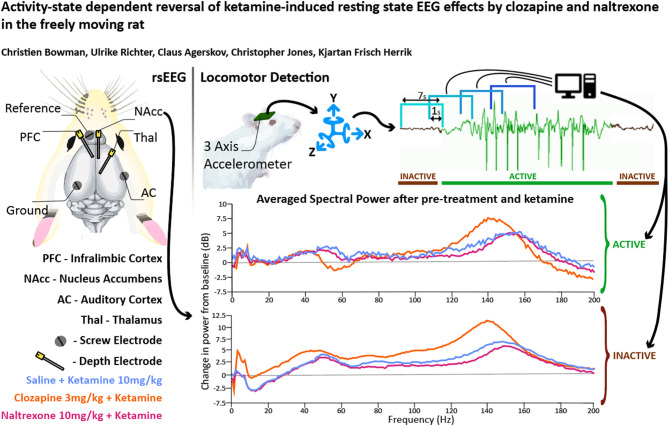

## Introduction

Ketamine is a non-competitive N-Methyl-D-aspartate receptor (NMDAR) antagonist investigated for its psychotomimetic properties (1, 2) and has, among other NMDAR antagonists, been used to model positive, negative and cognitive symptoms of schizophrenia (SZ) (3, 4). More recently, ketamine has gained attention for its robust, long-lasting, rapid-acting antidepressant (RAAD) effects (5, 6). The mechanism of therapeutic effect remains un-elucidated and understanding RAAD pathways is complicated by ketamine's affinities to receptors in opioid, norepinephric, dopaminergic and serotonergic systems (1, 7, 8).

Concerns that ketamine RAAD effects are opioid dependent were raised (9–13) after publication of two human studies using naltrexone (opioid antagonist) and ketamine (14, 15). Williams' study reported that naltrexone pre-treatment completely prevented ketamine RAAD improvements but left dissociation intact. Yoon's study found the opposite, but differed substantially in methodology. Subsequent research in rodents both implicates and refutes opioid involvement in the RAAD effect of NMDAR antagonists (9, 16–18). Debate remains as to whether acute naltrexone administration prevents RAAD effects, but further research in human subjects is stymied by ethical concerns.

*In vivo* local field potentials (LFP), electrocorticography (ECoG) and electroencephalography (EEG) are regularly used in translational research of disorders and potential therapeutics including Major Depressive Disorder (19–23). Despite the potential utility of these techniques to clarify the ketamine-opioid debate, at the time of writing no LFP or EEG data of acute ‘naltrexone plus ketamine' have been published.

LFP and ECoG paradigms are also translationally informative for schizophrenia (SZ) (20, 24, 25). Compared to healthy controls, unmedicated patients with SZ often present depressed activity between 7.5 and 20 Hz (26–31) and increases in higher bands > 30 Hz (24, 31–34). NMDAR antagonists including ketamine are used to model positive, negative and cognitive symptoms of this disorder (3, 4). After ketamine administration, rodents (35–40), healthy human volunteers (41–47) and unmedicated patients with SZ (48) all exhibit EEG disturbances similar to those seen in SZ patiesnts vs. healthy controls. In animal studies, where it is easier to record higher frequencies without interference from the skin and skull as in human s subjects, profound increases to high frequency oscillations (HFO [130–160 Hz]) are the most significant change reported (35–40, 49). In rodent studies in which locomotor states were tracked and separated with video tracking, ketamine-induced power spectra are distinctly different (50). The most clinically efficacious neuroleptic, clozapine, is effective in reducing positive and negative symptoms of SZ (51–53) and is known to modulate ketamine-induced spectral amplitudes (35, 36, 38, 40), however its efficacy at ameliorating induced power across different locomotor states is unknown.

Our research goals were to: apply an accelerometer-based behavioural detection method during LFP recordings to separate behavioural states and see if LFP profiles differed between them; identify if ketamine-induced LFP is modulated by naltrexone, a combination which is ethically problematic to study further in humans; and to investigate whether new LFP biomarkers of the most efficacious antipsychotic could be observed if recording data is behaviourally segregated; in particular the bands most disturbed by ketamine exposure: low Beta and HFO.

We characterised how LFP and ECoG spectra are modulated during ketamine exposure with and without pre-administration of naltrexone or clozapine. We recorded drug-induced LFP/ECoG in freely moving rats from four brain structures relevant to schizophrenia and major depressive disorder: LFPs from the thalamus (54–60), prefrontal cortex (PFC) (61–64), the nucleus accumbens (NAc) (65–69), and ECoG above the auditory cortex (AC) (70–76). To control for behavioural states, data from head-mounted accelerometers were utilised to algorithmically define if the animal was active or passive in each LFP/ECoG window. Additionally, to investigate whether neuroleptic effects on power spectra are occluded by behavioural artefacts, we employed the same paradigm with clozapine and ketamine. Freely moving rats were recorded during pre-treatment with either naltrexone or clozapine, ketamine challenge and pre-treatment with naltrexone or clozapine followed by ketamine challenge.

## Materials and Methods

### Materials

#### Subjects

Male Wistar rats (*n* = 115, 270–300 g, Charles River, Germany), were housed in cages with sawdust bedding and environmental enrichment (plastic shelter, gnawing blocks and paper strips) with food and water ad-libitum. Temperature and humidity were controlled and a 12:12 h reversed cycle (lights off at 6:00 AM) was implemented. All experiments were time matched and began at 09:00, during the lights off cycle in order to capture naturalistic wake behaviour. During the “lights off” period, red light was used to facilitate handling of animals. Animal welfare and weight recording was carried out daily.

Experimental procedures, animal housing and care were carried out in accordance with the Danish legislation according to the European Union regulation (directive 2010/63 of 22 September 2010), granted by the Animal Welfare Committee, appointed by the Ministry of Environment and Food of Denmark.

#### Drugs

Naltrexone (Lundbeck, 12 mg/ml) was diluted in 0.9% saline solution and administered subcutaneously (SC) at 1, 3 and 10 mg/kg; clozapine (Novartis, 10 mg/ml) was diluted with 0.5% methylcellulose was administered SC at 0.3, 1, and 3 mg/kg; ketamine (Ketolar, 50 mg/ml, Sigma) was diluted with 0.9% saline and administered SC at 10 mg/kg; Vehicle (VEH) control was 0.9% saline solution.

Rat pharmacologically relevant doses and timing to peak effect of pre-treatment were estimated on the basis of a review of the literature (35, 77–84) in conjunction with application of the “Human Effective Dose conversion formula” (85) in reverse to existing human study data in which the combination of naltrexone plus ketamine have been evaluated (14, 86). Ketamine dose was determined through extensive in-house studies (unpublished) and literature (37, 87) which demonstrate profound modulation of LFPs at 10 mg/kg.

#### Electrodes and Accelerometer

Custom accelerometers were manufactured by Ellegaard Systems and cables by PlasticsOne. Summed accelerometer output [equal to sqrt (X^2^ + Y^2^ + Z^2^)] was amplified (Precision Model 440; Brownlee, Palo Alto, CA, USA). Each of the 4 recording boxes with their own accelerometer and amplifier were calibrated to ensure equal output.

Depth electrodes (8IE3633SPCXE, E363-3-SPC, Elec.005-125MM SS, 25MM Length) and 6-way pedestals were purchased from PlasticsOne, manufactured by Bilaney Consultants GMBH.

### Methods

#### Surgical Procedure

Animals were habituated to placebo rimadyl pellets (Rimadyl MDs, BioServ, Flemington USA) 5 days prior to surgery. On the day of surgery, rats were anaesthetised with 0.25–0.3 ml/100 g subcutaneous (S.C.) injection of 1:1 hypnorm/dormicum and mounted in a stereotaxic frame (David Kopf Instruments, Tujunga, CA, USA) with blunt ear bars. marcain (0.2 ml s.c.) was injected under the scalp, and gel (Neutral Opthta Eye Gel) put on the eyes.

**Figure 1:**
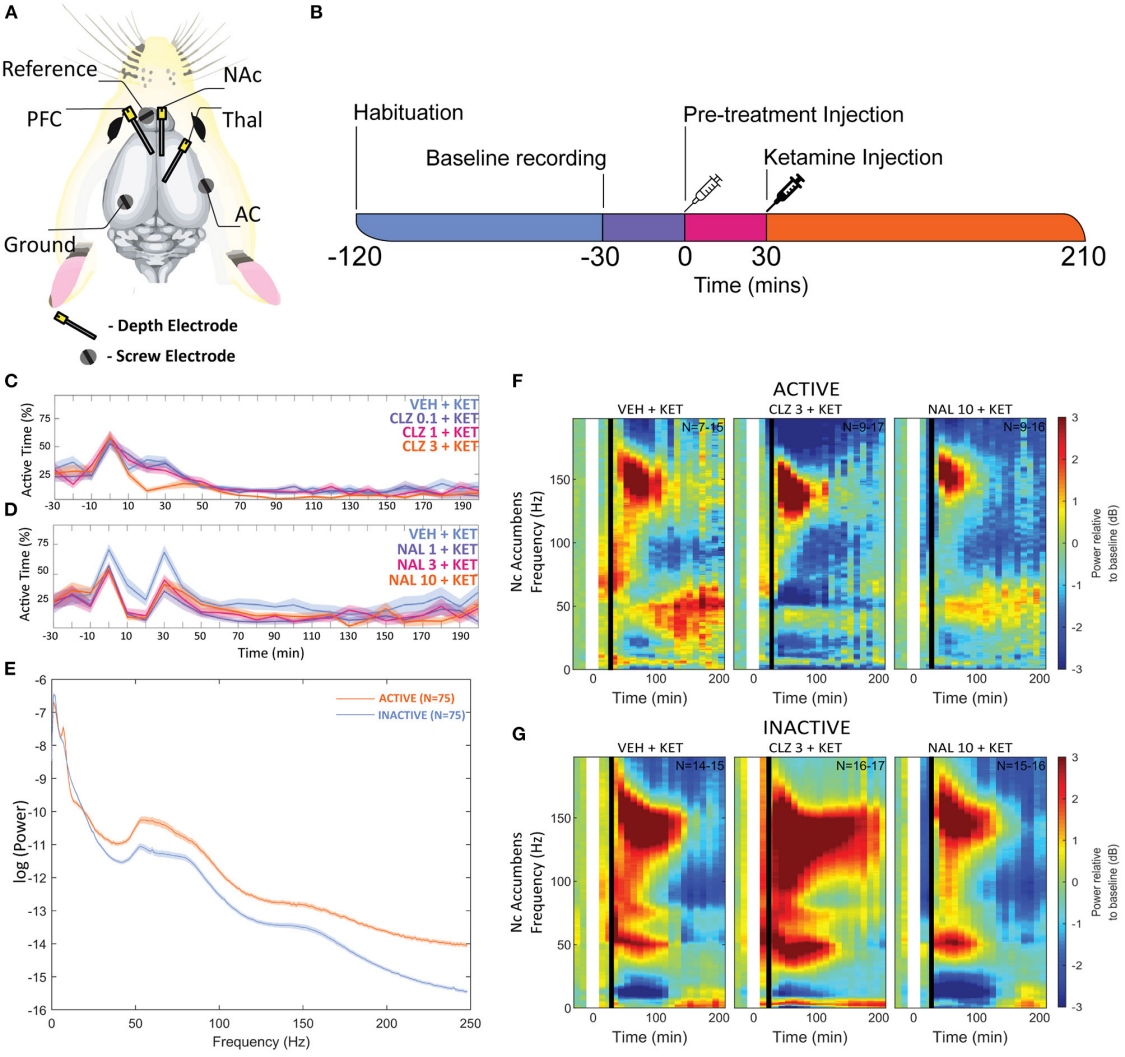
**(A)** Overview of electrode placements. **(B)** Diagram of experimental procedures in one recording session. Plots depicting the mean proportion of time spent in Active state for Clozapine **(C)** and Naltrexone **(D)** groups. Pre-treatment doses given in mg/kg. **(E)** A plot of mean baseline power in the Active and Inactive state taken at the NAc between “-30” and “0” min between 0 and 250 Hz. **(F,G)** Heatmaps depicting grand mean LFP [0–200 Hz] for an exemplar brain region, the NAc, for animals given VEH+VEH, VEH+KET, NAL+KET, and CLZ+KET during Active **(F)** and Inactive **(G)** epochs. Pre-treatment doses given in mg/kg. The first (leftmost, evenly green) timebin at the start of each plot indicates the baseline recording, to which the rest of the session was normalised. The white vertical bar at time 0 represents pre-treatment injection, and the black bar at time 30 indicates KET injection. Colours indicate change (in dB, which is logarithmic) to baseline. Number of subjects is given as a range (lowest-highest *n* subjects included in a timebin) in the top right of each plot; group sizes were equal, however not all subjects were included in all timebins as inclusion was conditional on 1) histological validation of electrode placement and 2) sufficient time spent in the Active or Inactive state in any given timebin. Full heatmaps for other regions can be found in Supplementary Information. VEH, saline; KET, ketamine 10 mg/kg; CLZ, clozapine 3 mg/kg; NAL, naltrexone 10 mg/kg.

Holes were drilled in the skull for three depth electrodes (Figure 1A) (E363-series; Invivo1/PlasticsOne, Roanoke, VA, USA) in the right infralimbic PFC (AP: +3.0 mm and ML:−0.7 mm from bregma, DV:−3.0 mm from the skull surface), Nucleus Accumbens shell (AP: +1.6 mm and ML: +1.0 mm from bregma and DV:−6.8 mm from the skull surface) and thalamus (AP:−2.8 mm and ML: +0.7 mm from bregma, DV:−4.4 mm from the skull surface) and three screw electrodes (E363-series, 15 mm, Invivo1/PlasticsOne, Roanoke, VA, USA) at vertex (AP:−5.0 mm and ML: +5.0 mm from bregma), auditory cortex (AP:−4.8 mm and ML:−6.4 mm from bregma) and a reference electrode (AP: +8.0 mm and ML: −2.0 mm from bregma). Ends of depth electrodes were cut before use to create an exposed tip. During the procedure, the rat's nails were trimmed to prevent grooming damage to surgical site.

Rats received 0.3 ml each of Norodyl and Noromox SC during the procedure, were placed under a warming lamp for 4 h and provided extra muesli. Rats were closely observed for 10–14-days recovery, sutures removed after 7–10 days. No rats lost >10% pre surgery weight. Animals received rimadyl pellets twice a day for 5 days.

Up until surgery, rats were maintained a normal 12 hr light cycle (lights on at 0600) so that surgery could be performed in full light without disturbing the rat's circadian rhythm. After surgery, the light cycle was reversed (lights off 0600) and 21 days was allowed to elapse between experimental recording in order to allow rats to fully acclimatise.

Rats were anaesthetised with sevoflurane and 0.1 mV passed through the electrodes to create a lesion for histological validation of depth electrode placement. Rats were then decapitated, whole brains extracted, and the brains were placed in labelled, protective bags and frozen at −80°C until cryosectioning. On the day of placement validation, frozen brains were cut at the transverse fissure with a scalpel to remove the cerebellum and mounted with polyethylene glycol & alcohol (OCT Tissue Tek®, Sakure, The Netherlands) to a metal stand, placed in a cryostat (Leica CM3050 S) and 20 μm slices of the lesion sites were taken for examination with an optical microscope. Data from electrodes placed outside of NAc, PFC or thalamus was discarded.

#### Groups

The rats were split into three groups:

Group 1 (*n* = 50) received VEH + ketamine (10 mg/kg), clozapine (0.3, 1, and 3 mg/kg) + ketamine (10 mg/kg). Each rat was dosed twice (different treatments) following a pseudo-randomised schedule that balanced for drug doses and order with at least 7 days of washout in between to prevent cumulative tolerance.

Group 2 (*n* = 50) received VEH + VEH, VEH + ketamine (10 mg/kg), naltrexone (1, 3, and 10 mg/kg) + ketamine (10 mg/kg). Each rat was dosed twice (different treatments) following a pseudo-randomised schedule that balanced for drug doses and order with at least 7 days of washout in between to prevent cumulative tolerance.

Group 3 (*n* = 15) received clozapine (0.3, 1 mg/kg) or naltrexone (1, 3, 10 mg/kg) to quantify peak plasma and brain concentrations.

#### EEG Recording

To facilitate habituation, rats were handled and placed individually into their respective EEG monitoring cage (Acrylic, 30 x 45 x 55 cm) within an electrically shielded, sound-proof box (90 x 55 x 65 cm) for at least 8 h (in <2-h sessions) in the week preceding experimental recording. during habituation, animals were connected to the EEG recording wire with the equipment switched off. Strict sound discipline was observed within the lab, preparation of drugs was performed under conditions that minimised disturbance sound.

On the days of recording, rats were placed into the cage, attached to a 6-pin recording wire on a rotating swivel and allowed to habituate for 120 min. A plastic spring (2.5 cm long when compressed and 2.5 cm diameter) was affixed to the rotating swivel and the recording wire affixed to the spring to allow 5 cm between the base of the cage and the terminal end of the wire. This alleviated the weight stress on the animal, allowed for vertical flexibility and prevented excess wire impeding animal movement. After 90 min of habituation to the recording environment, EEG and accelerometer recording began to establish a 30-min baseline for each session (Figure 1B). After the 30-min baseline recording, animals received a pre-treatment bolus of VEH (saline 0.9%), naltrexone (1, 3 or 10 mg/kg) or clozapine (0.3, 1, or 3 mg/kg) via SC flank injection.

Thirty minutes after pre-treatment, the animals received SC ketamine challenge (10 mg/kg) or VEH. Thirty minutes was selected as the optimal time for pre-treatment(s) to become effective following review of the literature (35, 77–84) and extensive in-house studies (unpublished). Recording of ECoG, LFP, and accelerometers continued for an additional 180 min after which animals were returned to their home cage.

Analogue LFP/ECoG signals were amplified (Precision Model 440; Brownlee, Palo Alto, CA, USA) and converted to a digital signal (CED Power 1401, Power 1 (625 k Hz, 16 bit) and CED Expansion ADC16; CED, Cambridge, England) at a sampling rate of 1 k Hz. LFP/ECoG signals were band-pass filtered at 0.01–300 Hz. Spike2 was used to simultaneously record inputs from microelectrodes, cameras and accelerometers, this ensured synchronised timestamps across file types.

#### Behavioural State Classification

Animal behaviour was recorded in parallel with a video camera and an accelerometer (custom-made with ADXL335Z, Analogue Devices) during each recording session. The accelerometer was fixed inside the plastic docking connector at the terminal end of the recording tether which screws onto the thread of the rodent's electrode headstage. The recorded video was used to qualify whether the animal was active or inactive, the latter here being defined as a state with no visible body movement with the exception of occasional micromovements of the nose and head. The accelerometer signal was then reviewed in parallel with the video recording, and an *ad hoc* threshold for distinguishing activity from inactivity was determined. For further processing the signal was smoothed with a gaussian kernel and divided into 7-s segments bins with 1-s overlap. If the signal during a segment was above the threshold for at least 60% of the time the segment was determined to be from an active period, correspondingly if the signal was below the threshold for at least 60% of the time the segment was determined to be from an inactive period. Segments that fulfilled neither criteria were left unclassified.

#### Data Analysis

This study was intended to test whether the investigated drugs and behavioural states affect LFP and ECoG signals. This hypothesis was measured by consideration of EEG profiles across the following frequency bands: Delta (0.1–4 Hz), Theta (4–10 Hz), low Beta (10–20 Hz), high Beta (20–30 Hz), low Gamma (30–60 Hz), high Gamma (60–130 Hz), HFO (130–160 Hz), and Ultra High Frequency Oscillations UHFO (160–200 Hz) separated by behavioural state. To avoid power line interference, 2-Hz sections of frequency centred at 50, 100, and 150 Hz were excluded from analysis.

Analysis was carried out in MATLAB (MathWorks, Natick, MA). Signals were divided into consecutive 2-s segments with 1-s overlap. To minimise influence of artefacts, 2-s segments in which the signal exceeded ± 7 standard deviations (SD) from the mean were excluded from analysis. Furthermore, through comparison with the outcome of the behavioural state classification, each 2-s segment was assigned to either the active or inactive motor state or left unclassified. Next, a spectrogram with time and frequency resolution of 1 s and 0.5 Hz, respectively, was produced for each brain area by applying the Fast Fourier transform (FFT) to each 2-s segment. A spectrogram is a time series of power spectral densities and allows assessment of the spectral content of a signal over time, such as the presence of oscillatory activity in certain frequency bands.

When analysing the raw power, the logarithm was taken, otherwise each power spectral density was normalised to the baseline by dividing with the average power spectral density during the stable 30-min baseline period immediately prior to injection. The baseline-normalised spectral content was then converted to decibel (dB). Next, the power spectral densities were averaged over non-overlapping consecutive 10-min bins, positioned such that the time of injection is at 0 min, thereby producing spectrograms with 10-min time resolution. The steps of baseline normalisation and 10-min averaging were done both disregarding the behavioural state as well as only considering power spectral densities from segments classified as active or inactive, respectively. As a final step, grand averages were produced for each combination of brain area, behavioural state and treatment group.

Statistical analysis was conducted for averages over certain time intervals (10–30 min for pre-treatment, 40–70 min for ketamine challenge) and/or the already outlined frequency bands (see Tables 1–**3**). To investigate whether there were any significant treatment effects compared to the VEH + ketamine group, repeated measures analysis of variance (RM-ANOVA) was performed using MATLABs fitglme function with subsequent multiple comparison correction using Tukey's honest significant difference (HSD). *P* < 0.05 were considered significant. The fitted generalised linear mixed effects (GLME) model included an intercept and a factor for the treatment group, as well as a random-effects intercept for each animal to account for animal-specific variations. If applicable (i.e., when averaging only over a time interval or frequency band), the model also included a factor for the frequency/time bin and its interaction with the treatment group.

**Table 1:** Tables of averaged power spectra between 40 and 70 min of experimentation for “Inactive” and “Active” epochs of animals given vehicle pre-treatment (at 0 min) + vehicle or ketamine (10 mg/kg at 30 min).

**Region**	**Dose**	**Active**								**Inactive**							
**Ketamine 40–70 min**																	
		0–4	4–10	10–20	20–30	30–60	60–130	130–160	160–200	0–4	4–10	10–20	20–30	30–60	60–130	130–160	160–200
NAc	V	0.43	0.90	0.21	0.30	0.31	0.75	−0.13	−0.13	0.20	−0.64	−0.43	0.19	0.60	1.12	1.03	1.10
	V+K	0.49	0.87	−0.59	−0.79	0.81	0.64	3.67	0.78	−0.38	−1.72	−3.67	−1.45	2.08	2.24	6.09	2.51
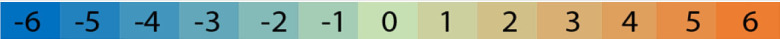 Baseline-normalised power (db)																	

*Values are given in normalised dB change from baseline of each session. dB is a logarithmic scale, meaning that “−3dB” = 50% of original value, whilst “3dB” = 200% of original value. Values significantly different vs. vehicle are coloured according to the valence of change from baseline. Full tables of p-values and non-segregated 'Any' spectra can be found in Supplementary Information. Dose is given in mg/kg; V, Vehicle; K, ketamine 10 mg/kg*.

For each recording, the time the animal spent in the active and inactive behavioural state, respectively, was also calculated during non-overlapping 10-min bins, and grand averages were calculated for each treatment group. Statistical differences were assessed similar as for the spectral power in a certain frequency band, i.e., by using a GLME model with an intercept, a factor for the treatment group and the time interval and their interaction, and random-effects intercept for each animal, followed by Tukey's HSD. An animated visualization of the fundamental principles behind LFP recording, our recording procedure and some of the locomotor state differences is provided in the Supplementary Material.

#### Drug Exposure Determination

To determine if the selected doses of naltrexone, clozapine and ketamine resulted in translationally relevant concentrations in the blood and brain of subjects, a drug exposure study was performed. Satellite animals (*n* = 3 per dose per drug) were treated by subcutaneous (SC) injection with Clozapine (0.3, 1, or 3 mg/kg) or Naltrexone (1, 3, or 10 mg/kg) then terminal venous blood and whole brain samples were taken at 1 h for exposure determination. In brief, plasma was isolated from whole blood and whole brains were isolated according to a previously described protocol (87). The brain tissue was prepared for extraction by dilution in buffer (1:5 w/v in deionised water) then homogenised by isothermal focused acoustic ultra-sonication using a Covaris instrument [Covaris E220x, 3.5 min at a bath temperature of 7°C with a peak power of 500 W and average power of 250 W (1,000 cycles per burst, duty cycle 50%)].

Total drug concentrations (Naltrexone or Clozapine) were determined in plasma and brain samples using high performance liquid chromatography coupled with tandem mass spectrometry (LC-MS/MS). The plasma (25 μL) and brain homogenate (25 μL) samples were precipitated with acetonitrile (4 volumes), centrifuged (3,500 g, 20 min, 5°C) and the supernatant (50 μL) diluted with water (3 volumes) before injection on the LC-MS/MS system. Drug concentrations were determined from calibration lines of known concentrations spiked into control plasma or brain homogenate and extracted under identical conditions. Bioanalysis was performed using a Waters Aquity UPLC coupled to a Waters XevoTQXS detector. A Waters Acquity UPLC HSS C18 SB, 1.7 μm, 30 × 2.1 mm column was used operating at 40°C. Mobile phase A consisted of 0.1% Formic Acid in water and mobile phase B of 0.1% Formic Acid in Acetonitrile. The LC flow rate was 0.6 mL/min. Analytes were separated on the LC column using a gradient. From 0 to 0.5 min the gradient was held at 2% mobile phase B. From 0.5 to 2 min B changed from 2 to 95% and was held at 95% until 2.5 min. Thereafter, between 2.5 and 2.7 min, B changed to 2% and was held at 2% from 2.7 to 4 min. Electrospray ionisation-MS (ESI-MS) was performed in positive MRM mode. For ketamine, clozapine, and naltrexone the parent:daughter [M+H]^+^ ions: 327.09^+^→ 270.08^+^ and 342.17^+^→ 270.15^+^ were selectively monitored for quantification, respectively.

## Results

LFPs were similarly modulated by each drug combination across all recorded brain structures. Thus, in the interests of space and clarity, figures and tables in the manuscript are restricted to the Active and Inactive state in an exemplar region, the NAc, as this is where ketamine's effects are frequently the most profound in both our study and the wider literature (35, 36, 39, 66). The full figures and tables for each brain structure, activity state and un-separated LFP data may be found in the Supplementary Information.

### Locomotor State Globally Alters Local Field Potentials

To control for animal behaviour during freely moving rsEEG, recorded epochs (2 s) were separated by locomotor activity level. This produced separate Active and Inactive baseline-corrected data for each 10-min timebin. Active or Inactive state was defined by a two-state classifier using data from a 3-axis, head-mounted accelerometer. Experimental animals were Inactive >50% in all conditions, and passivity increased towards the end of each recording session. Animals were transiently more active after injections at 0 and 30 min, however pre-treatment with Naltrexone (1, 3, and 10 mg/kg, dose dependent relationship) and clozapine (3 mg/kg) abolished this (Figures 1C,D). No hyperlocomotion was observed in any pre-treatment conditions after ketamine challenge (30 min).

Separating LFP by locomotor activity revealed activity-state-specific changes to spontaneous neural activity. Power in Active epochs was higher in all but Delta and low Beta bands (Figure 1E). In addition, a peak in baseline Theta amplitude is observed only in the Active state. Some compound induced changes were occluded entirely by analysing Active and Inactive LFP together (Supplementary Figures 1, 2 and Supplementary Tables 1–9). Pharmacologically-induced spectra were more pronounced during inactivity – mixed modelling of dB change from baseline found that Activity State significantly predicted magnitude of change from baseline (*F*_1,9_ = 138.20; *p* < 0.0001). Differences between Active and Inactive were confirmed with a *post hoc* investigation using Tukey's HSD (*p* < 0.0001).

### Ketamine Suppresses Beta, Enhances HFO

After ketamine administration (30 min), rats pre-treated with saline displayed broad depression of frequencies below 30 Hz, barring Theta [4–10 Hz] in the Active PFC. These effects were more pronounced during Inactive epochs with few exceptions. Beta power [10–30 Hz] was suppressed by ketamine at all recording electrodes and across all activity states. Low beta [10–20 Hz] underwent the most profound depression in the Inactive thalamus and AC [4.39 and 4.99 dB decrease vs. baseline, respectively].

By contrast, ketamine induced increased power in frequencies 30–160 Hz. Inactive HFO [130–160 Hz] was subject to the most robust increase in oscillatory power, brain wide and across both motor states. Of note, the magnitude of HFO power during Inactive epochs [3.69–6.09 dB increase from baseline] did not overlap with the range during Active [1.22–3.67 dB increase from baseline]. In particular, the NAc (Figures 1F,G) and PFC recorded the most robust increases to spectral power.

### Effect of Clozapine on Spontaneous Power Spectra

#### Pre-treatment

Clozapine pre-treatment elicited oscillatory activity throughout the recording regions during Inactive epochs. Interestingly, the mid-dose (1 mg/kg) induced Inactive LFP power across the broadest range of frequency bands and brain areas (Figures 1F,G, Table 2 and Supplementary Tables 2, 3). Clozapine dose dependently increased Delta [0-4 Hz] activity in the Inactive Thalamus, PFC, and most substantially in the AC (*p* = 0.0006; *p* = 0.004; *p* = 0.0009). During Inactivity, clozapine (1 and 3 mg/kg) substantially enhanced spectral power in frequency bands between 30 and 60 Hz and across all electrodes.

**Table 2:** Table of averaged power spectra at 10–30 min.

**Region**	**Dose**	**Active**								**Inactive**							
**Clozapine 10–30 min**																	
		0–4	4–10	10–20	20–30	30–60	60–130	130–160	160–200	0–4	4–10	10–20	20–30	30–60	60–130	130–160	160–200
NAc	V+K	0.63	1.20	0.60	0.41	0.72	0.94	−0.07	−0.05	0.77	0.27	0.20	0.47	0.80	0.94	0.46	0.45
	0.3	0.14	1.39	0.98	0.43	0.17	0.71	−0.13	−0.54	1.28	0.11	−0.25	0.53	1.42	1.82	1.39	1.37
	1	−0.26	0.82	0.15	−0.09	0.33	0.58	0.05	−0.18	2.02	0.87	0.46	1.28	2.43	2.35	1.95	1.83
	3	−1.14	0.27	−0.47	−0.21	0.13	0.66	−0.11	−0.78	1.57	0.43	0.01	0.69	2.25	2.01	1.71	0.95
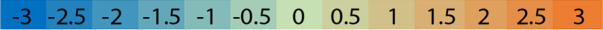 Baseline-normalised power (db)																	

*Pre-treatment with Clozapine was given at 0 mins. Separated by Active (left) and Inactive (right) epochs. Values are given in dB change from baseline. dB is a logarithmic scale, meaning that “−3dB” = 50% of original value, whilst “3dB” = 200% of original value. Values that are significantly different vs. vehicle + ketamine are coloured according to the valence of change from baseline. Full tables of p-values and non-segregated “Any” spectra can be found in Supplementary Information. Dose is given in mg/kg; V, Vehicle; K, ketamine 10 mg/kg*.

Clozapine's effects on Active spectra were primarily depressive. In the Active PFC and Thalamus, activity in several frequency bands (low and high beta [10–20 Hz; 20–30 Hz] and low *y* [30–60 Hz]) were depressed by clozapine (3 mg/kg). Suppression in Active epochs was eclipsed when analysing both motor states.

#### After Ketamine Challenge

Clozapine largely reversed ketamine's effects on lower bands, and enhanced effects >60 Hz. Ketamine induced depression of Theta [4–10 Hz] was completely ameliorated by clozapine in the Inactive state. In low beta [10–20 Hz], where ketamine induced suppression was more profound, clozapine partially returned LFP power towards baseline throughout the AC, PFC and Thalamus (3 mg/kg: *p* = 0.0006; *p* = 0.0007; *p* = 0.0009) (Supplementary Tables 6, 7). In the AC for example, ketamine depressed low beta to 40.18% of baseline, and 3 mg/kg clozapine returned this to 84.14% of baseline. A similar relationship, though of a lower magnitude, was also displayed in neighbouring frequency band high beta [20–30 Hz]. Reversal of beta suppression was exclusively seen in the Inactive state. By contrast, Active beta depression at the PFC and NAc (Table 3 and Supplementary Tables 6, 7) was exacerbated by clozapine (3 mg/kg) (*p* = 0.015; *p* = 0.005).

**Table 3:** Table of averaged power spectra at 40–70 min.

**Region**	**Dose**	**Active**								**Inactive**							
**Clozapine 40–70 min**																	
		0–4	4–10	10–20	20–30	30–60	60–130	130–160	160–200	0–4	4–10	10–20	20–30	30–60	60–130	130–160	160–200
NAc	V+K	−0.23	1.21	−0.45	−0.51	0.54	1.16	3.05	0.60	0.02	−1.48	−2.37	−0.82	1.63	1.77	4.63	1.31
	0.3	−0.15	0.64	−1.28	−1.54	−0.49	0.31	4.47	−0.92	0.91	−0.29	−3.02	−0.42	2.92	3.33	7.37	1.90
	1	−0.77	0.66	−1.82	−1.66	−0.53	0.25	4.61	−0.56	1.34	0.63	−2.15	−0.16	2.73	3.25	7.76	2.11
	3	−1.33	0.34	−1.77	−1.44	−0.58	0.65	5.97	−1.79	0.75	0.56	−1.73	0.10	2.78	3.90	8.51	0.93
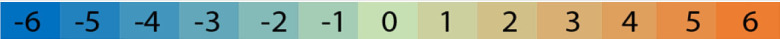 Baseline-normalised power (db)																	

*Pre-treatment with Clozapine was given at 0 min, and ketamine at 30 min. Separated by Active (left) and Inactive (right) epochs. Values are given in dB change from baseline. dB is a logarithmic scale, meaning that “−3dB” = 50% of original value, whilst “3dB” = 200% of original value. Values that are significantly different vs. vehicle + ketamine are coloured according to the valence of change from baseline. Full tables of p-values and non-segregated “Any” spectra can be found in Supplementary Information. Dose is given in mg/kg; V, Vehicle; K, ketamine 10 mg/kg*.

Ketamine-induced power in higher frequencies was synergistically enhanced by clozapine. Robust, dose-dependent increases were seen to ketamine-induced *y* [60–130 Hz] and HFO [130–160 Hz] in the Inactive NAc [3.05dB to 3.90dB, *p* = 0.00097; 4.63–8.51 dB, *p* = 0.00096, respectively]. Clozapine (3 mg/kg) also dose dependently reversed ketamine-induced depression of low *y* in the Active PFC, returning it almost to baseline. Analysis of LFP without separating by locomotor state rendered this effect invisible (Supplementary Table 3).

**Figure 2:**
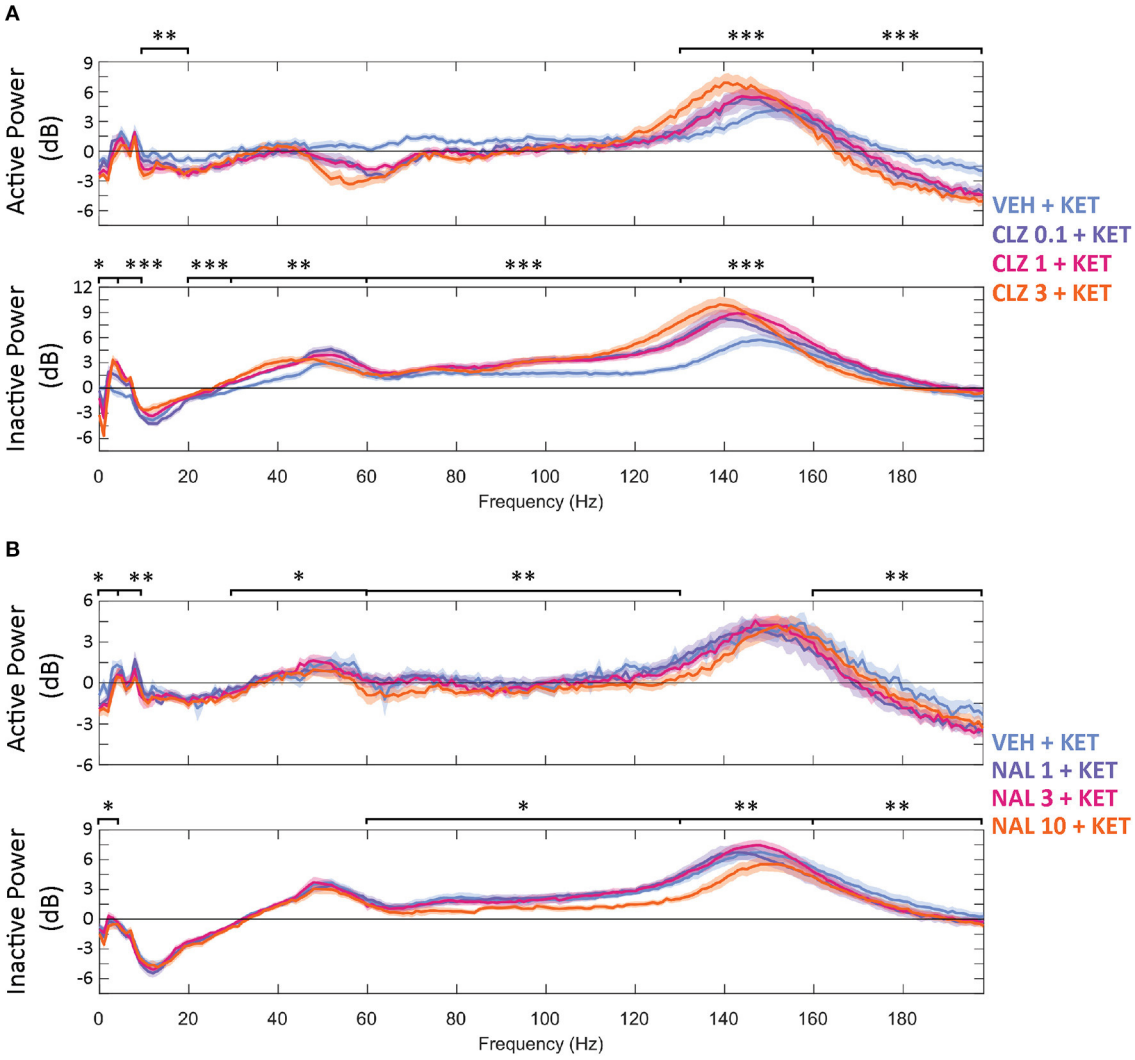
Baseline-normalised, averaged spectra recorded at the NAc of CLZ **(A)** and NAL **(B)** groups between 40 and 70 min (10 min after KET and 40 min after pre-treatment). Displayed in dB change from baseline. Legends give pre-treatment doses in mg/kg. Significant differences of pre-treatment + KET spectra vs. VEH = KET are indicated by *p < 0.05/**p < 0.01/***p < 0.001. VEH, saline; KET, ketamine 10 mg/kg; CLZ, clozapine; NAL, naltrexone.

Increasing doses of clozapine also modulated the peak frequency of ketamine-induced spectra in the NAc. Clozapine increased peak power, but downshifted HFO peak frequency [from 151 to 143 Hz] and low *y* [58 Hz to 51 Hz] (Figure 2A). Interestingly, clozapine dose and peak HFO exhibit a biphasic relationship – 1 mg/kg clozapine peak HFO was higher than either 0.3 or 3 mg/kg. The nadir of beta suppression was also downshifted by clozapine, from 18 to 15 Hz.

### Effect of Naltrexone on Spontaneous LFP Spectra

#### Pre-treatment

Naltrexone (time 0) reduced oscillatory power globally in the acute pre-treatment phase (10–30 min) across a broad range of frequency bands (Figures 1F,G, Table 4 and Supplementary Tables 4, 5). Naltrexone decreased Inactive high beta [20–30 Hz] power and a biphasic relationship was seen between dose strength, with the mid dose (3 mg/kg) inducing the greatest depression [NAc, *p* = 0.001; PFC, *p* = 0.006; Thalamus, *p* = 0.0008]. Increasing doses of naltrexone depressed all frequency bands >30 Hz during Inactive epochs and across all electrodes. Active HFO power was also reduced below baseline at every electrode (10 mg/kg/Active; AC, *p* = 0.0007; NAc, *p* = 0.0009; PFC, *p* = 0.04; Thalamus, *p* = 0.0009).

**Table 4:** Table of averaged power spectra at 10–30 min.

**Region**	**Dose**	**Active**								**Inactive**							
**Naltrexone 10–30 min**																	
		0–4	4–10	10–20	20–30	30–60	60–130	130–160	160–200	0–4	4–10	10–20	20–30	30–60	60–130	130–160	160–200
NAc	V+K	0.45	0.58	0.25	−0.16	0.29	0.67	−0.21	−0.13	0.50	−0.06	−0.34	0.27	0.97	1.21	0.39	1.20
	0.3	0.08	0.52	0.35	0.63	0.88	0.67	−0.29	−0.49	−0.18	−1.17	−0.99	−0.37	0.27	0.51	0.39	−0.01
	1	−0.57	0.73	−0.13	0.14	0.53	0.17	−0.78	−0.89	−0.15	−1.11	−1.08	−0.37	0.24	−0.11	−0.18	−0.36
	3	−0.70	−0.03	−0.24	0.13	0.39	−0.89	−1.26	−1.15	−0.34	−0.63	−0.38	−0.06	0.13	−1.83	−2.01	−1.67
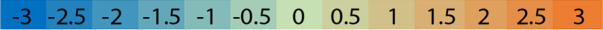 Baseline-normalised power (db)																	

*Pre-treatment with Naltrexone was given at 0 min. Separated by Active (left) and Inactive (right) epochs. Values are given in dB change from baseline. dB is a logarithmic scale, meaning that “−3dB” = 50% of original value, whilst “3dB” = 200% of original value. Values that are significantly different vs. vehicle + ketamine are coloured according to the valence of change from baseline. Full tables of p-values and non-segregated “Any” spectra can be found in Supplementary Information. Dose is given in mg/kg; V, Vehicle; K, ketamine 10 mg/kg*.

#### After Ketamine Challenge

Naltrexone pre-treatment did not significantly alter ketamine-induced beta depression in the Inactive or Active state (Table 5 and Supplementary Tables 8, 9). Non-modulation of low beta [10–20 Hz] was consistent at all electrodes and states (10 mg/kg: Inactive: AC, *p* = 0.99/; NAc, *p* = 0.99; PFC, *p* = 0.99; Thalamus, *p* = 0.39; Inactive: AC, *p* = 0.77; NAc, *p* = 0.19; PFC, *p* = 0.83; Thalamus, *p* = 0.74). In bands *y* and above, naltrexone reduced ketamine-induced power in the Inactive PFC (10 mg/kg, low *y, p* = 0.006; high *y, p* = 0.001; HFO, *p* = 0.0009; UHFO, *p* = 0.007), though the resulting LFP power remained substantially higher than baseline. Similar, but less consistent suppression was observed at other electrodes, and during Active epochs (Supplementary Tables 8, 9).

**Table 5:** Table of averaged power spectra at 40–70 min.

**Region**	**Dose**	**Active**								**Inactive**							
**Naltrexone 40–70 min**																	
		0–4	4–10	10–20	20–30	30–60	60–130	130–160	160–200	0–4	4–10	10–20	20–30	30–60	60–130	130–160	160–200
NAc	V+K	0.45	0.58	0.25	−0.16	0.29	0.67	−0.21	−0.13	0.50	−0.06	−0.34	0.27	0.97	1.21	0.39	1.20
	0.3	0.08	0.52	0.35	0.63	0.88	0.67	−0.29	−0.49	−0.18	−1.17	−0.99	−0.37	0.27	0.51	0.39	−0.01
	1	−0.57	0.73	−0.13	0.14	0.53	0.17	−0.78	−0.89	−0.15	−1.11	−1.08	−0.37	0.24	−0.11	−0.18	−0.36
	3	−0.70	−0.03	−0.24	0.13	0.39	−0.89	−1.26	−1.15	−0.34	−0.63	−0.38	−0.06	0.13	−1.83	−2.01	−1.67
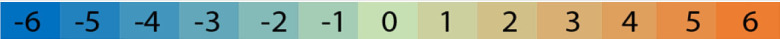 Baseline-normalised power (db)																	

*Pre-treatment with Naltrexone was given at 0 min, and ketamine at 30 min. Separated by Active (left) and Inactive (right) epochs. Values are given in dB change from baseline. dB is a logarithmic scale, meaning that “−3dB” = 50% of original value, whilst “3dB” = 200% of original value. Values that are significantly different vs. vehicle + ketamine are coloured according to the valence of change from baseline. Full tables of p-values and non-segregated “Any” spectra can be found in Supplementary Information. Dose is given in mg/kg; V, Vehicle; K, ketamine 10 mg/kg*.

The width of peak HFO that ketamine affected was also modulated by naltrexone. Animals pre-treated with saline saw significant ketamine-induced power in a moderate band [135–167 Hz], 1 mg/kg naltrexone widened the band of affected frequencies by 43.8% [121–167 Hz] vs. saline, whilst 10 mg/kg naltrexone thinned affected HFO 84.4% [152–157 Hz] vs. saline (Figure 2B).

### Quantification of Clozapine and Naltrexone Concentrations in Satellite Animals

Drug concentrations were determined in satellite animals (*n* = 3 per dose per drug) and are presented in Table 6. Both drugs distributed to the brain with total brain to plasma ratios ~3.9 and 21, respectively. Ketamine exposures were not assessed in order to avoid animal handling causing interference during the pharmacodynamic measurement window. The ketamine SC dose was selected based on data from several rat cognitive pharmacology models (data not presented). The Cmax in these studies confirmed consistent plasma and brain ketamine exposures were achieved following 10 mg/kg SC administration (mean total plasma concentration at 0.5 h post dose = 951 ng/mL (range 670–1,311 ng/mL; *n* = 5 studies), brain: plasma total concentration ratio at 0.5 h post dose = 3.6).

**Table 6:** Clozapine and Naltrexone concentrations measured in terminal plasma and brain homogenate samples 1 h after subcutaneous injection (*n* = 3 satellite animals).

**Drug pre-treatment**	**Clinical dose (mg)**	**Back translated rat dose (mg/kg)**	**SC Dose (mg/kg)**	**Time point (h)**	**Total plasma concentration; Mean ± SD (ng/mL)**	**Total brain concentration; Mean ± SD (ng/mL)**	**Total brain: plasma concentration ratio (Kp); Mean ± SD**
Clozapine	12.5	1.29	0.3	0.5	12.4 ± 1.7	268 ± 35	22 ± 1.3
			1	0.5	55 ± nv	1322 ± nv	13 ± nv
			3	0.5	112 ± 12	3055 ± 421	27 ± 1
Naltrexone	25–50	3.875	1	0.5	69 ± 5	305 ± 31	4.4 ± 0.2
			3	0.5	221 ± 33	883 ± 48	4.0 ± 0.5
			10	0.5	875 ± 125	2899 ± 227	3.4 ± 0.5

## Discussion

The primary findings of this study are: (1) the effect on LFP/ECoG power of clozapine, ketamine and naltrexone depends on locomotor state; (2) ketamine-induced beta suppression in the Inactive state is reversed by the antipsychotic clozapine but is preserved during naltrexone co-administration; and (3) broadband ketamine induced enhancement of higher frequencies, especially HFO, is bolstered by clozapine but dampened by naltrexone.

### Locomotor State Separation

The two-state classifier revealed locomotor-state specific effects on LFP amplitudes that otherwise would have been occluded, validating head mounted accelerometers as an alternative to video-tracking solutions. More sophisticated machine learning solutions utilising both LFP and accelerometers can detect up to 7 behaviours (88), but may not be suitable for every study i.e.,: when recording from different brain structures than the original study. Non-invasive head-mounted accelerometers are compatible with any freely moving recording paradigm (EEG, 2-photon calcium microscopy, etc.) and require 0.008% as much data storage when compared to video files from the same recording session. As substantial differences in spontaneous brain activity exist between locomotor states, seen previously (37) and in the present study, it is imperative that efficient and economical behavioural segregation of freely moving experimentation is implemented in future studies.

Separating locomotor states highlighted Active state spectra that were obscured when looking at non-classified LFP epochs summed together. The Active-state peak in baseline Theta has some precedent: Theta power is known to spike during exploratory behaviour in rodents (89, 90) and more recently was observed to increase in walking human subjects (91). During pharmacological manipulations, Active spectra were generally outweighed due to 1) the inclination of rats in this study to remain passive >50% of the recording session in all groups and pharmacological conditions; and 2) pharmacologically induced changes to spontaneous Inactive power were of a substantially larger magnitude. As neuronal firing increases during movement in response to increased sensory input and processing (37, 92), we hypothesise that the smaller pharmacological deviations in Active vs. Inactive results from 1) circuits modulated by clozapine/ketamine/naltrexone are also engaged during locomotion, thus baseline Active LFPs are closer to physiological maximum and pharmacological enhancement above baseline is limited; or 2) distinct circuits of neurons engaged during Active behaviour generate spectral activity that outweighs LFPs generated by modulation of drug-susceptible circuits. In support of the former proposition, comparing raw baseline power showed that Active power was almost exclusively higher than Inactive (Figure 1E). Investigation of LFP properties of specific neural circuits exclusively during movement is required to elucidate the degree to which either hypothesis is responsible.

We did not observe significant ketamine-induced hyperlocomotion in any compound combination. This is concurrent with other observations in rats given 10 mg/kg ketamine (37, 39) but is contrary to other studies using 2.5–10 mg/kg (40, 93, 94). Habitation differences between studies reporting hyperlocomotion may explain this: rats habituated to the recording box for 90 min in this study before recording of EEG or locomotor activity began, vs. 60 min (94) and 30 min to room/0 min to arena (40, 93). We primarily suspect that this study's decision to employ a reversed light cycle may be responsible. This decision was made to allow rats to be recorded during their usual waking hours (as in human rsEEG) to capture the most translatable data. As animals in the present study had already been awake for several hours (experiments started at 0900, 3 h after “lights out”) their level of wakefulness may have been higher than rats in other studies recorded during the light phase (when they are naturally inclined to sleep). Ketamine (2.5–10 mg/kg) delays onset of sleep (95) and this may be interpreted as induction of hyperactivity during the light phase.

Irrespective of hyperlocomotion, the importance of separating LFP data by activity state is clear from our report. Developing user friendly systems capable of automatically detecting three or more behaviours may improve the reliability of spectral activity studies even further. Controlling for motor activity is certain to be a building block in bridging the translation gap between pre-clinical and clinical research.

### Beta Suppression and Psychotomimetic Features

Beta band suppression could indicate manifestation of psychomimetic properties of ketamine. We observed that beta amplitudes were depressed by ketamine during Inactive epochs, and that the antipsychotic clozapine dose dependently reversed this. Clinical findings are strikingly resemblant to our own: low beta is found to be depressed in unmedicated schizophrenic patients (26, 27, 30) as are EEG spectra between [7.5–12.5 Hz] (termed alpha in human EEG studies, overlapping with low beta [10–20 Hz]) (30). Both low beta disturbances and symptoms measured by the Positive and Negative Symptoms Scale (PANSS) are reduced by acute and chronic clozapine treatment (29). Moreover, suppression of low beta during ketamine exposure has been correlated with symptom severity as scored by the Clinician Administered Dissociative States Scale (CADSS) (43, 44) and other purpose-built self-report questionnaires (47) when administered to healthy subjects. Finally, in one study that failed to find significance between CADSS scores and ketamine induced low beta suppression, it was found that restoration of low beta by midazolam and improvement in dissociation scores in CADSS were causally linked (46). These results dovetail with the presence and absence of low beta suppression reported in our study; suppression occurs during psychotomimetic drug exposure, while clozapine ameliorates this. Importantly, these human EEG studies were performed in an “Inactive”-like state i.e.,: 10 min of eyes closed sitting still—and we only saw reversal of ketamine induced effects on beta in this state, which may explain why it has not received attention in preclinical studies until now.

Behavioural measures follow a similar pattern. Positive, negative and cognitive symptoms were inhibited by administering clozapine to human patients with SZ (53, 96–99), even when given ketamine (48). Ketamine-induced cognitive deficits are also prevented in mice by clozapine administration (100). Naltrexone did not change the dissociative aspects of acute ketamine exposure in Williams (2019) study, and the same combination of compounds produced no changes in beta in this study. The results of this study contribute more evidence towards an association between beta depression at rest and dissociative symptoms. Reversal of beta suppression may prove to be a useful preclinical biomarker for assessing neuroleptics.

### Higher Frequencies

#### Clozapine and Ketamine Enhances HFO Power Through Asynchrony

In agreement with previous locomotor-state-separated EEG analyses (37), power in frequencies above 30 Hz were broadly enhanced by ketamine, particularly in the Inactive state. Drug effects in the gamma band largely resemble those in HFO albeit with a lower magnitude, therefore as in other NMDAR antagonist LFP studies (35, 36, 38, 39, 49) we focus the discussion on effects in the HFO band. Ketamine induced-HFO were further strengthened by clozapine across both locomotor states. Increased HFO power can represent asynchronous activity in several distinct local neuronal populations, and/or circuit(s) that have become dysregulated (101, 102). Such asynchrony was indicated by the broader peak of spectral power/greater spectral entropy observed with increasing doses of clozapine in the present study (101, 103). Whilst it could be hypothesised that circuit desynchronisation occurs from clozapine (104) and ketamine (2) possessing opposing affinities for NMDAR on GABAergic interneurons, it has been demonstrated that the firing rate of local GABAergic interneurons in the rat thalamus and PFC are not significantly altered by ketamine (87). Ketamine potentially drives HFO through increased firing of excitatory pyramidal neurons (105–107). According to the “direct” hypothesis, ketamine-induced, NMDAR-dependent plasticity-related protein synthesis seen in pyramidal neurons (108, 109) is responsible for increased excitatory drive (107, 110).

Clozapine has affinities for several receptors that could recruit additional neuronal populations, generating more power yet less synchrony in the HFO band compared to ketamine alone. Agonism at NMDAR on local GABAergic interneurons, known generators of fast rhythmic activity in their own right (106), is one example. Clozapine additionally increases the firing of dopaminergic neurons in the ventral tegmental area by 100% (111), which innervates two structures this study observed broadband HFO increases within: the PFC (112) and NAc (113). However, single unit electrophysiology studies are necessary to characterise the precise neuronal sub-populations that are recruited during acute ketamine and clozapine exposure vs. ketamine alone.

#### Naltrexone Modulates Ketamine Induced Excitatory Disinhibition

Our findings indicate a clear difference in LFPs between ketamine, and ketamine plus naltrexone; a combination that is suspected to block RAAD effects (11, 14). While ketamine's RAAD effects are suspected to be driven through transient excitation of pyramidal neurons and synaptogenesis in key brain structures such as the PFC (114–119), the precise mechanistic pathway(s) through which improvement manifests is not yet fully elucidated. In addition, mechanisms have been identified through which opioid blockade could prevent RAAD (120) including BDNF upregulation and synaptogenesis (121), which is blocked by naltrexone (122); and acute agonism at mu-opioid receptors situated on neurons in the lateral habenula, dorsal raphe nucleus and ventral tegmental area. Inhibition of these neurons, *via* ketamine's antagonism at NMDAR and agonism at mu-opioid receptors, triggers downstream disinhibition of serotonergic and dopaminergic neurons in the PFC and NAc (120, 123–127). In this proposed circuit, as increasing doses of naltrexone block mu-opioid receptor agonism by ketamine, less excitatory disinhibition manifests in the PFC and NAc. Accordingly, we report a dose-dependent decrease of ketamine-induced HFO in these locations. If future studies confirm that naltrexone blocks ketamine's RAAD properties, increased HFO in the PFC and NAc should prove to be valuable biomarkers for antidepressant drug research.

Whilst naltrexone and clozapine had opposite effects in this band, it is important to be cautious drawing direct comparisons between the two until more acute studies have been conducted. One important limitation of this study is the exclusion of behavioural outcome measures for depressive and psychotomimetic symptoms. Thus, we can only say that in drug combinations that block RAAD effects in humans, we see suppression of ketamine induced HFO. Investigation in human subjects and in pre-clinical depression models to characterise the relationship between HFO amplitudes and RAAD effects is recommended.

## Concluding Remarks

This is the first study to investigate differences in locomotor state ketamine LFP induced by the neuroleptic clozapine and the opioid antagonist naltrexone. Our results reveal distinct profiles of LFP activity across locomotor states and demonstrate the pressing need to separate these for accurate analysis in future studies. Separating out Activity states stands to make translational research more directly comparable to human data. We also show powerful modulation of ketamine LFPs by clozapine and naltrexone. Potent reversal of beta suppression by clozapine exclusively during the Inactive state hints at its potential value as a biomarker for neuroleptic efficacy. We also establish here for the first time that HFO is materially different between ketamine with/without naltrexone pre-treatment, and the relationship we document here aligns with the proposed outcomes of a previously proposed pathway through which ketamine's RAAD effects are impacted by opioid blockade. Our findings in both beta and HFO bands appear to support literature describing opioid involvement in ketamine's therapeutic mechanism. Future acute studies in humans with these compounds will help tease out the intricate dance between LFP and subjective, symptomatic changes. Both HFO and beta may prove to be invaluable biomarkers in the hunt for more efficacious antidepressant and neuroleptic medications with milder side effects.

## Data Availability Statement

The raw data supporting the conclusions of this article will be made available by the authors, without undue reservation.

## Ethics Statement

The animal study was reviewed and approved by Experimental Procedures, Animal Housing and Care were carried out in accordance with the Danish legislation according to the European Union Regulation (directive 2010/63 of 22 September 2010), granted by the Welfare Committee, appointed by the Ministry of Environment and Food of Denmark.

## Author Contributions

CB: study design, writing, pilot data collection, graphical abstract, figure production, and data analysis. UR: data analysis, figure production, and writing. CA: data analysis and review. CJ: exposure study and writing. KH: study design, writing, direction, and review. All authors contributed to the article and approved the submitted version.

## Funding

Facilities and funding was provided by Lundbeck (Denmark). Additional funding was given in the form of an Erasmus+ grant for international research *via* Maastricht University. Sponsors did not influence study design.

## Conflict of Interest

The authors declare that the research was conducted in the absence of any commercial or financial relationships that could be construed as a potential conflict of interest.

## Publisher's Note

All claims expressed in this article are solely those of the authors and do not necessarily represent those of their affiliated organizations, or those of the publisher, the editors and the reviewers. Any product that may be evaluated in this article, or claim that may be made by its manufacturer, is not guaranteed or endorsed by the publisher.

## Abbreviations

AC: Auditory cortex
ECoG: Electrocorticography
EEG: Electroencephalography
FFT: Fast Fourier Transform
GABA: γ-aminobutyric acid
HFO: High frequency oscillations
LFP: Local field potential
PFC: Prefrontal cortex (human) / Infralimbic Cortex (rat)
NAc: Nucleus accumbens
NMDAR: N-Methyl-D-aspartate receptor
RAAD: Rapid acting antidepressant
rsEEG: Resting state EEG
S.C: Subcutaneous
SZ: Schizophrenia
VEH: Vehicle.
