# Supplementary material for: Activity-State Dependent Reversal of Ketamine-Induced Resting State EEG Effects by Clozapine and Naltrexone in the Freely Moving Rat
Source: Front Psychiatry. 2022 Jan 27;13:737295. doi: 10.3389/fpsyt.2022.737295 (PMC8830299; doi:10.3389/fpsyt.2022.737295)
Supplement: Supplementary file 1 [file Data_Sheet_1.docx]

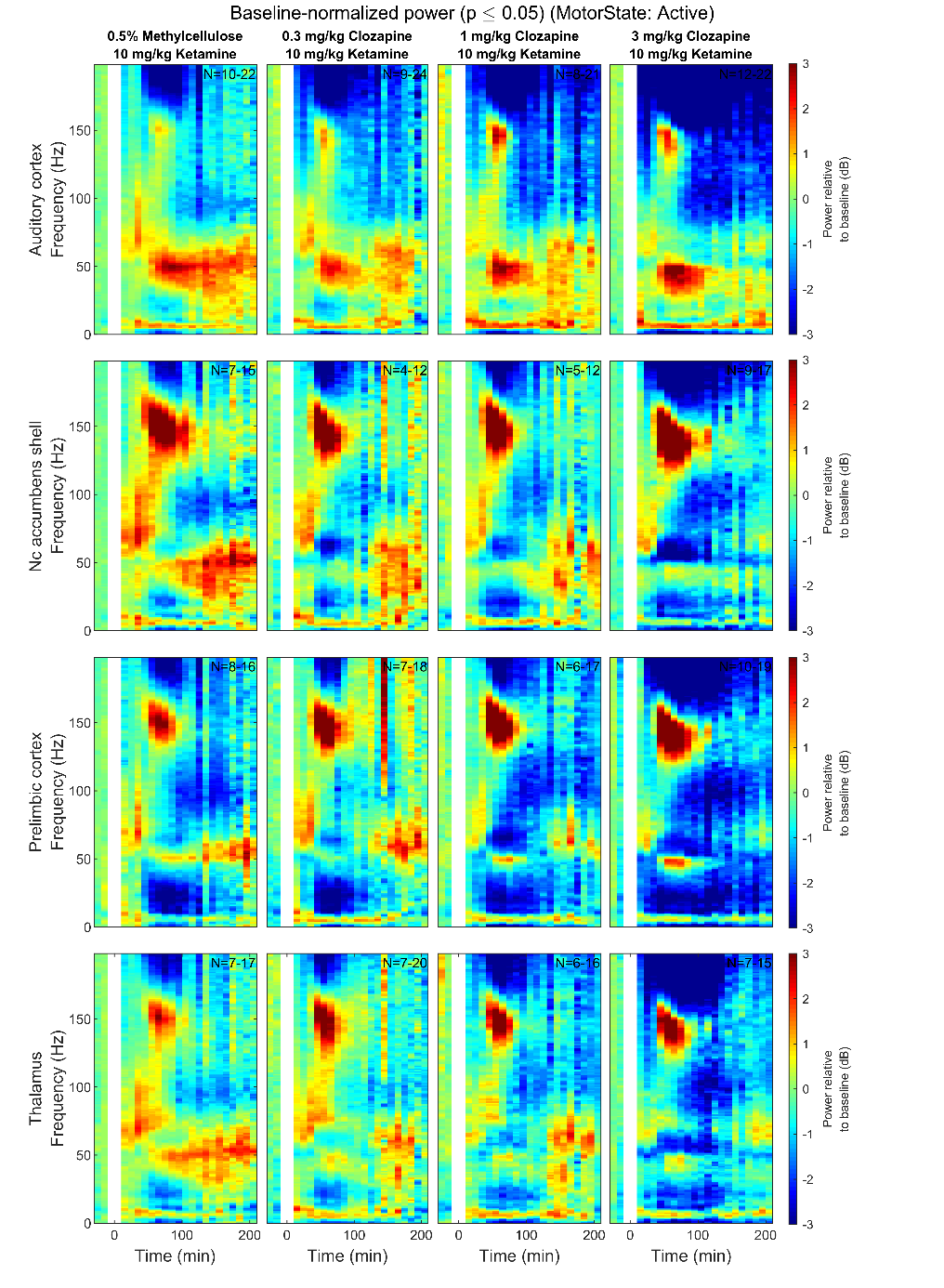

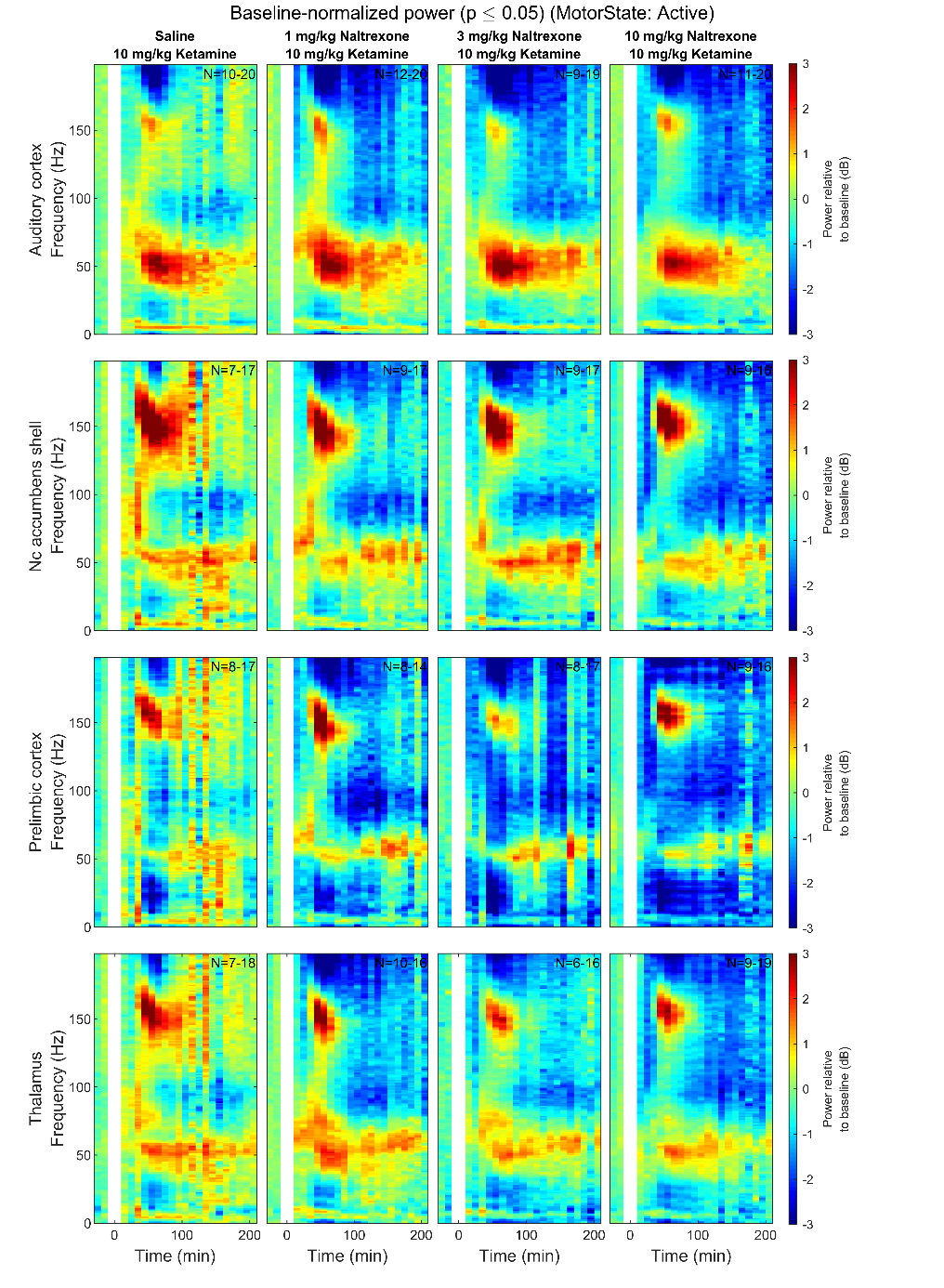


Supplementary Figure 1: Heatmaps depicting grand mean LFP [0-200 Hz] for the Auditory Cortex, Nucleus Accumbens, Prelimbic Cortex and Thalamus for animals given experimental compounds during Active epochs. The first (leftmost, evenly green) timebin at the start of each plot indicates the baseline recording, to which the rest of the session was normalized. Pre-treatment was given at 0 min (white bar) and ketamine injection was given at 30, indicated by the spike in HFO power. Number of subjects is given as a range (lowest-highest *n* subjects included in a timebin) in the top right of each plot; group sizes were equal, however not all subjects were included in all timebins as inclusion was conditional on 1) histological validation of electrode placement and 2) sufficient time spent in the Active or Inactive state in any given timebin. Colours indicate change (in dB, which is logarithmic) to baseline. *Hz = Hertz, dB = decibel*


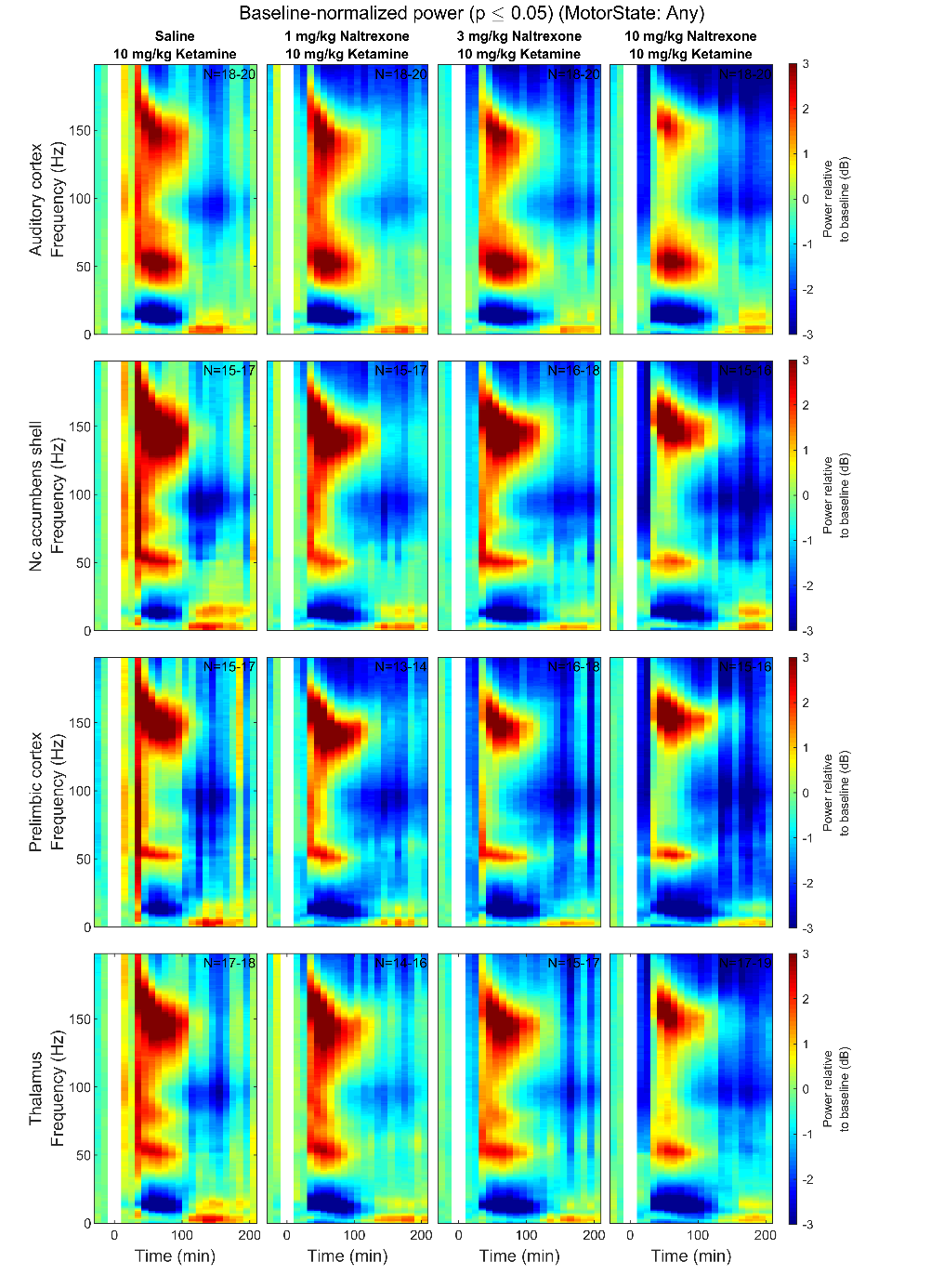

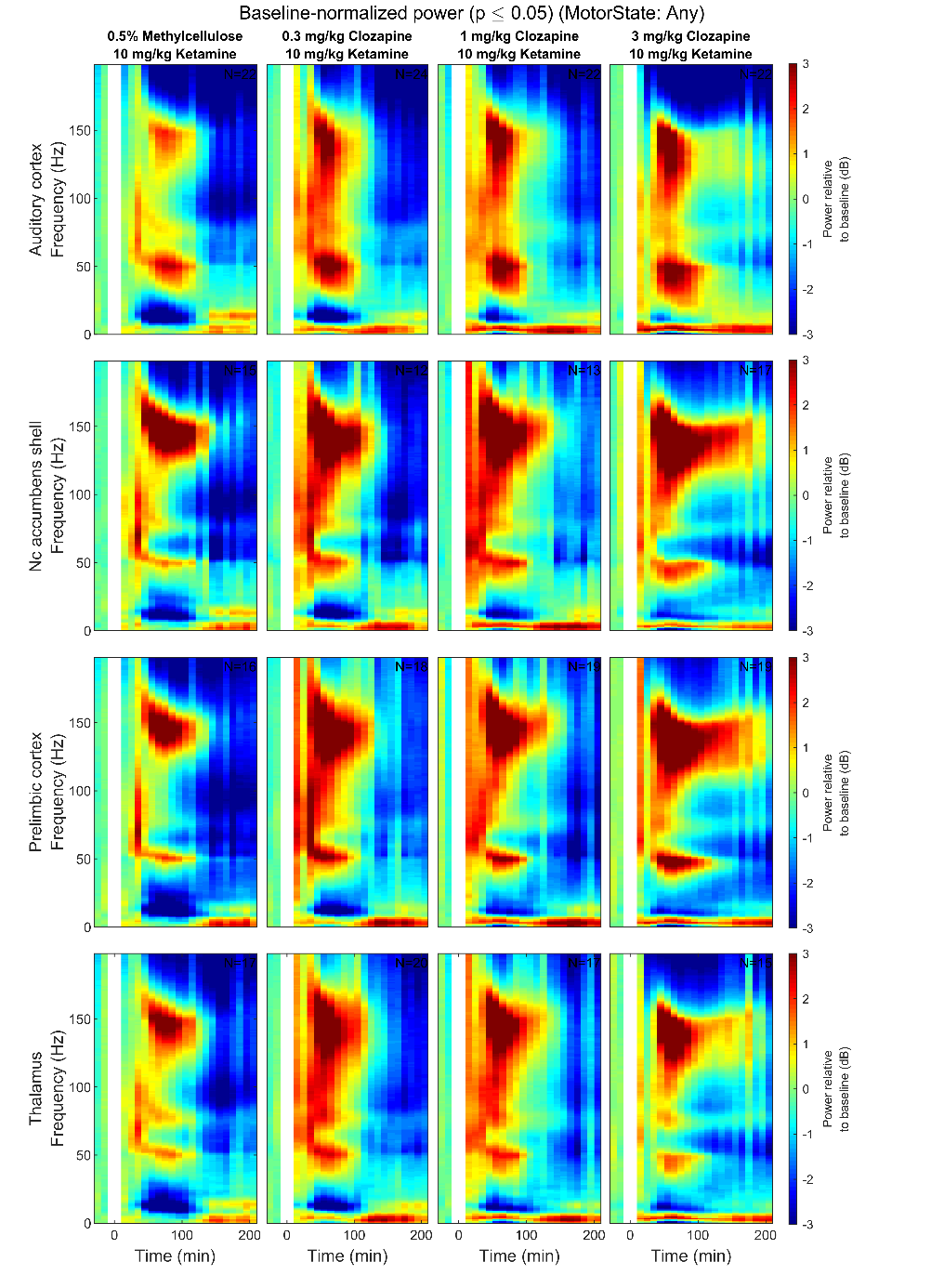


Supplementary Figure 2: Heatmaps depicting grand mean LFP [0-200 Hz] for the Auditory Cortex, Nucleus Accumbens, Prelimbic Cortex and Thalamus for animals given experimental compounds during all epochs (no locomotor sorting). The first (leftmost, evenly green) timebin at the start of each plot indicates the baseline recording, to which the rest of the session was normalized. Pre-treatment was given at 0 min (white bar) and ketamine injection was given at 30, indicated by the spike in HFO power. Number of subjects is given as a range (lowest-highest *n* subjects included in a timebin) in the top right of each plot; group sizes were equal, however not all subjects were included in all timebins as inclusion was conditional on 1) histological validation of electrode placement and 2) sufficient time spent in the Active or Inactive state in any given timebin. Colours indicate change (in dB, which is logarithmic) to baseline. *Hz = Hertz, dB = decibel*


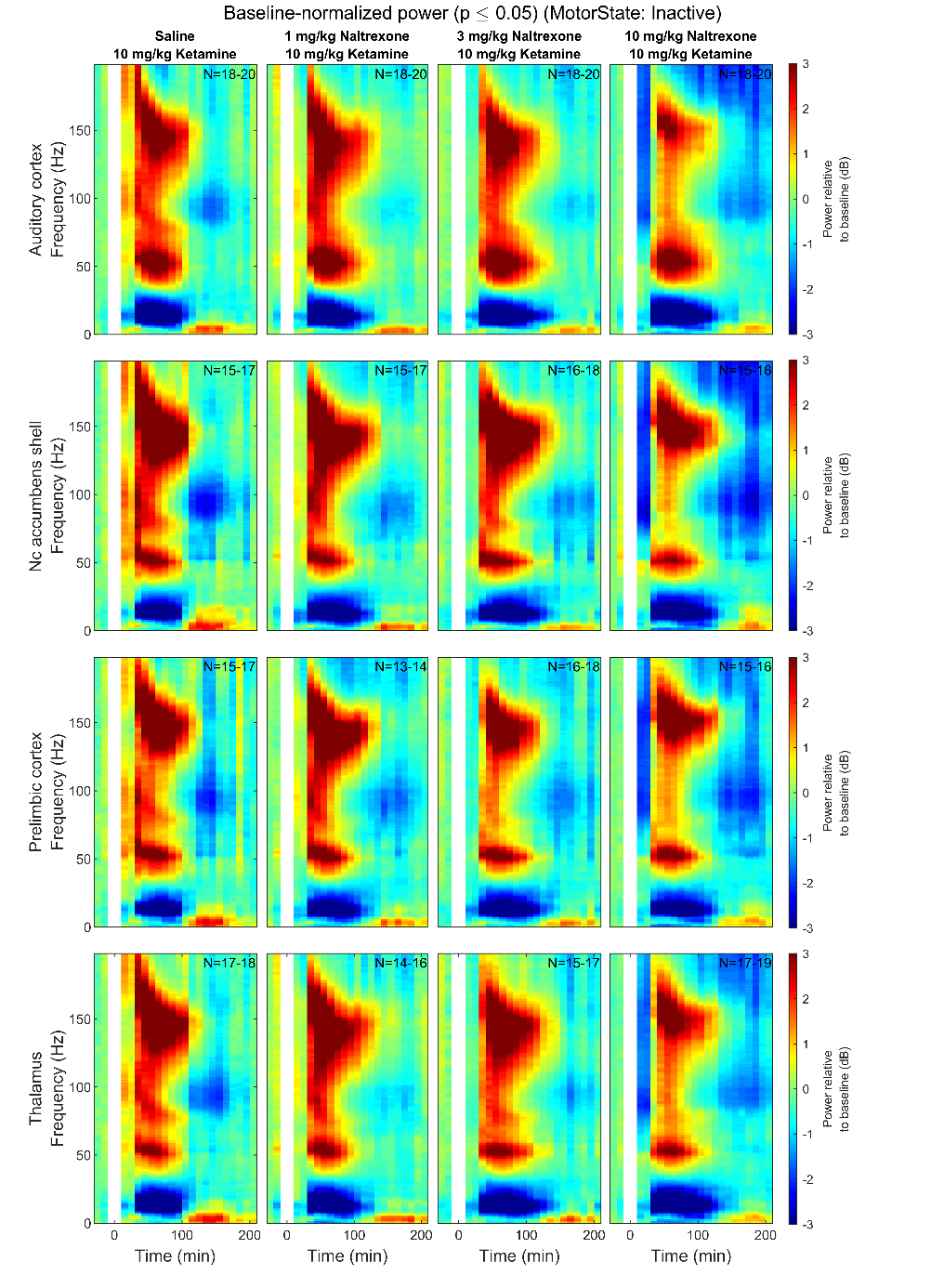

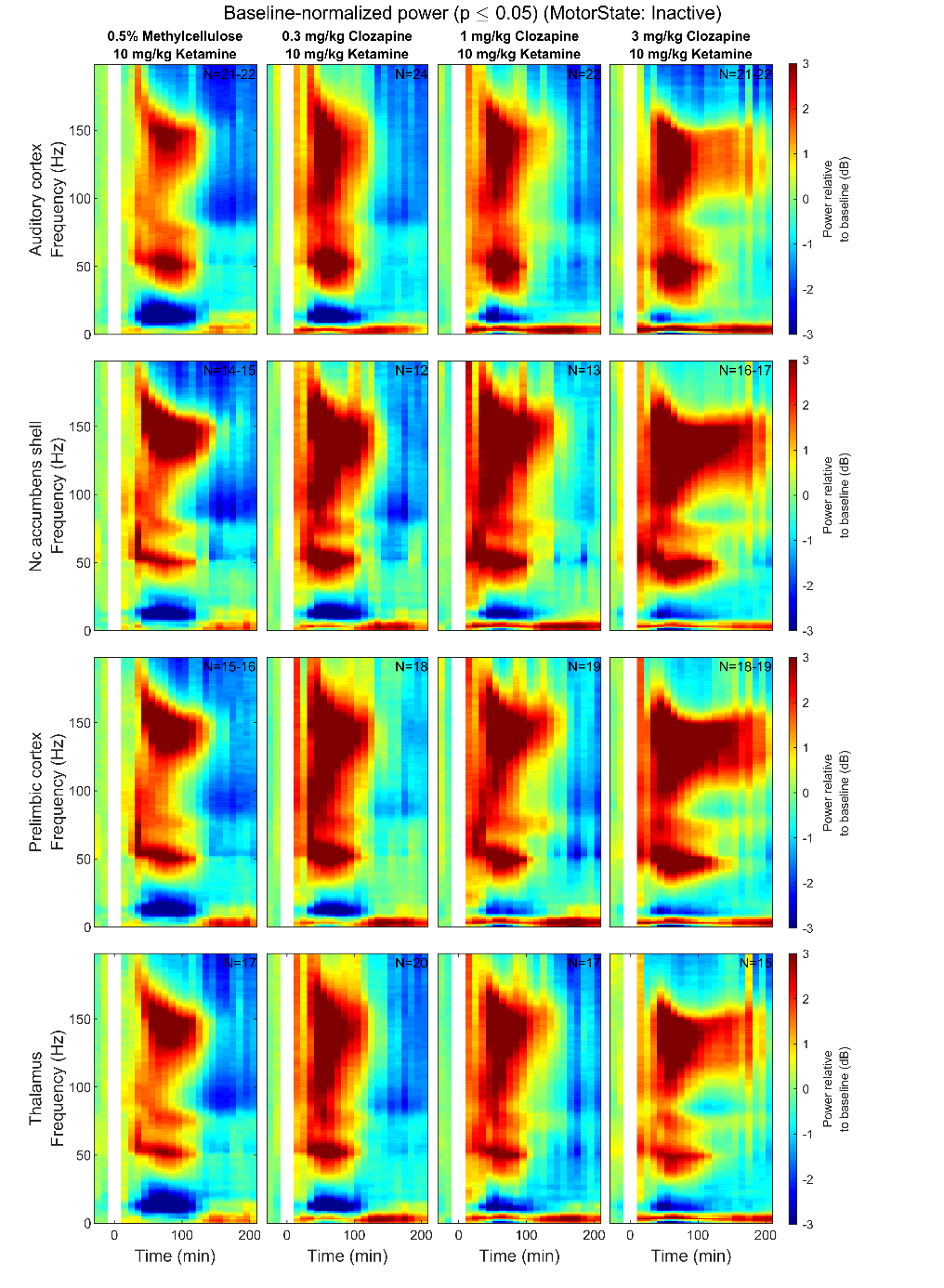


Supplementary Figure 3: Heatmaps depicting grand mean LFP [0-200 Hz] for the Auditory Cortex, Nucleus Accumbens, Prelimbic Cortex and Thalamus for animals given experimental compounds during Inactive locomotor epochs. The first (leftmost, evenly green) timebin at the start of each plot indicates the baseline recording, to which the rest of the session was normalized. Pre-treatment was given at 0 min (white bar) and ketamine injection was given at 30, indicated by the spike in HFO power. Number of subjects is given as a range (lowest-highest *n* subjects included in a timebin) in the top right of each plot; group sizes were equal, however not all subjects were included in all timebins as inclusion was conditional on 1) histological validation of electrode placement and 2) sufficient time spent in the Active or Inactive state in any given timebin. Colours indicate change (in dB, which is logarithmic) to baseline. *Hz = Hertz, dB = decibel*

Supplementary Table 1: Tables of averaged power spectra (top) and p values (bottom) between 40-70 minutes of experimentation for ‘Active’ (left), ‘Any’ (middle) and ‘Inactive’ (right) epochs of animals given vehicle pre-treatment (0 min) *+* vehicle or ketamine (10 mg/kg at 30 min). Values are given in normalized dB change from baseline of each session. dB is a logarithmic scale, meaning that “*-3* dB” = 50% of original value, whilst “3dB” = 200% of original value. Values significantly different vs vehicle are coloured according to the valence of change from baseline*.* P-values were determined as described in Methods 2.2.5 and are given to 3 decimal places *Dose is given in mg/kg;* *V = Vehicle; K = ketamine 10 mg/kg*

| **KETAMINE 40-70 MINS** | | | | | | | | | | | | | | | | | | | | | | | | | |
| --- | --- | --- | --- | --- | --- | --- | --- | --- | --- | --- | --- | --- | --- | --- | --- | --- | --- | --- | --- | --- | --- | --- | --- | --- | --- |
| Region | Dose | **ACTIVE** | | | | | | | | **ANY** | | | | | | | | **INACTIVE** | | | | | | | |
|  |  | 0-4 | 4-10 | 10-20 | 20-30 | 30-60 | 60-130 | 130-160 | 160-200 | 0-4 | 4-10 | 10-20 | 20-30 | 30-60 | 60-130 | 130-160 | 160-200 | 0-4 | 4-10 | 10-20 | 20-30 | 30-60 | 60-130 | 130-160 | 160-200 |
| AC | V | 0.06 | 0.34 | -0.09 | -0.03 | 0.18 | 0.50 | -0.20 | -0.18 | 0.26 | -0.17 | -0.51 | 0.03 | 0.36 | 0.80 | 0.47 | 0.41 | 0.44 | -0.47 | -0.52 | 0.10 | 0.46 | 0.89 | 0.88 | 1.03 |
|  | V+K | 0.01 | 1.05 | -0.74 | -0.58 | 2.08 | 0.67 | 1.22 | -1.42 | -0.28 | -1.46 | -3.77 | -1.32 | 2.39 | 1.55 | 2.78 | 0.18 | -0.29 | -2.43 | -4.99 | -1.83 | 2.68 | 2.11 | 3.69 | 1.26 |
| NAcc | V | 0.43 | 0.90 | 0.21 | 0.30 | 0.31 | 0.75 | -0.13 | -0.13 | 0.17 | 0.03 | -0.25 | 0.24 | 0.49 | 1.09 | 0.70 | 0.70 | 0.20 | -0.64 | -0.43 | 0.19 | 0.60 | 1.12 | 1.03 | 1.10 |
|  | V+K | 0.49 | 0.87 | -0.59 | -0.79 | 0.81 | 0.64 | 3.67 | 0.78 | -0.10 | -0.98 | -2.54 | -1.08 | 1.57 | 1.62 | 5.38 | 1.83 | -0.38 | -1.72 | -3.67 | -1.45 | 2.08 | 2.24 | 6.09 | 2.51 |
| PFC | V | 0.23 | 0.60 | -0.47 | -0.51 | -0.21 | 0.43 | -0.14 | -0.01 | 0.18 | 0.21 | 0.00 | 0.30 | 0.54 | 1.09 | 0.65 | 0.77 | 0.22 | -0.76 | -0.50 | 0.10 | 0.61 | 1.12 | 0.95 | 1.09 |
|  | V+K | -0.04 | 0.19 | -1.79 | -2.13 | -0.20 | -0.40 | 2.26 | 0.40 | -0.51 | -1.22 | -2.65 | -1.55 | 0.89 | 0.73 | 3.81 | 1.08 | -0.66 | -1.84 | -3.36 | -1.08 | 2.30 | 1.81 | 5.05 | 2.14 |
| Thalamus | V | 0.42 | 0.70 | 0.00 | -0.02 | 0.26 | 0.67 | -0.10 | -0.10 | 0.09 | 0.05 | -0.11 | 0.25 | 0.46 | 0.83 | 0.58 | 0.53 | 0.05 | -0.54 | -0.31 | 0.25 | 0.60 | 0.94 | 1.00 | 1.15 |
|  | V+K | 0.42 | 0.72 | -0.88 | -1.06 | 1.00 | 0.62 | 2.43 | -0.02 | -0.27 | -1.18 | -3.12 | -1.39 | 1.42 | 1.53 | 4.00 | 1.20 | -0.58 | -2.17 | -4.39 | -1.81 | 1.81 | 2.17 | 4.91 | 2.19 |
| 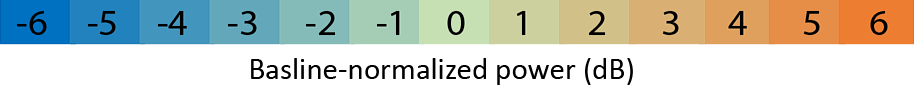 | | | | | | | | | | | | | | | | | | | | | | | | | |
| Region | Dose | **ACTIVE** | | | | | | | | **ANY** | | | | | | | | **INACTIVE** | | | | | | | |
|  |  | 0-4 | 4-10 | 10-20 | 20-30 | 30-60 | 60-130 | 130-160 | 160-200 | 0-4 | 4-10 | 10-20 | 20-30 | 30-60 | 60-130 | 130-160 | 160-200 | 0-4 | 4-10 | 10-20 | 20-30 | 30-60 | 60-130 | 130-160 | 160-200 |
| AC | V |  |  |  |  |  |  |  |  |  |  |  |  |  |  |  |  |  |  |  |  |  |  |  |  |
|  | V+K | 0.910 | 0.125 | 0.001 | 0.001 | 0.001 | 0.001 | 0.001 | 0.001 | 0.001 | 0.001 | 0.001 | 0.001 | 0.001 | 0.029 | 0.001 | 0.715 | 0.001 | 0.001 | 0.001 | 0.001 | 0.001 | 0.001 | 0.001 | 0.714 |
| NAcc | V |  |  |  |  |  |  |  |  |  |  |  |  |  |  |  |  |  |  |  |  |  |  |  |  |
|  | V+K | 0.079 | 0.341 | 0.013 | 0.002 | 0.133 | 0.756 | 0.001 | 0.001 | 0.353 | 0.006 | 0.001 | 0.001 | 0.001 | 0.001 | 0.001 | 0.066 | 0.109 | 0.009 | 0.001 | 0.001 | 0.001 | 0.001 | 0.001 | 0.001 |
| PFC | V |  |  |  |  |  |  |  |  |  |  |  |  |  |  |  |  |  |  |  |  |  |  |  |  |
|  | V+K | 0.001 | 0.001 | 0.001 | 0.001 | 0.001 | 0.001 | 0.001 | 0.528 | 0.001 | 0.001 | 0.001 | 0.006 | 0.549 | 0.598 | 0.001 | 0.716 | 0.001 | 0.018 | 0.001 | 0.002 | 0.002 | 0.253 | 0.001 | 0.112 |
| Thalamus | V |  |  |  |  |  |  |  |  |  |  |  |  |  |  |  |  |  |  |  |  |  |  |  |  |
|  | V+K | 0.315 | 0.960 | 0.001 | 0.001 | 0.001 | 0.621 | 0.001 | 0.426 | 0.001 | 0.002 | 0.001 | 0.001 | 0.002 | 0.073 | 0.001 | 0.334 | 0.001 | 0.001 | 0.001 | 0.001 | 0.001 | 0.002 | 0.001 | 0.113 |

Supplementary Table 2: Table of averaged power spectra at 10-30 mins. Pre-treatment with Clozapine was given at 0 mins. Separated by Active (left), Any (middle) and Inactive (right) epochs. Values are given in dB change from baseline. dB is a logarithmic scale, meaning that –3dB = 50% of original value, whilst 3dB = 200% of original value. Values that are significantly different vs ketamine are coloured according to the valence of change from baseline. *Dose is given in mg/kg;* *V = Vehicle; K = ketamine 10 mg/kg*

| **CLOZAPINE 10-30 MINS** | | | | | | | | | | | | | | | | | | | | | | | | | |
| --- | --- | --- | --- | --- | --- | --- | --- | --- | --- | --- | --- | --- | --- | --- | --- | --- | --- | --- | --- | --- | --- | --- | --- | --- | --- |
| Region | Dose | **ACTIVE** | | | | | | | | **ANY** | | | | | | | | **INACTIVE** | | | | | | | |
|  |  | 0-4 | 4-10 | 10-20 | 20-30 | 30-60 | 60-130 | 130-160 | 160-200 | 0-4 | 4-10 | 10-20 | 20-30 | 30-60 | 60-130 | 130-160 | 160-200 | 0-4 | 4-10 | 10-20 | 20-30 | 30-60 | 60-130 | 130-160 | 160-200 |
| AC | V+K | 0.31 | 0.96 | 0.18 | 0.06 | 0.29 | 0.27 | -0.53 | -0.69 | 0.69 | 0.30 | -0.07 | 0.00 | 0.19 | 0.32 | -0.19 | -0.42 | 0.98 | 0.28 | -0.16 | 0.08 | 0.41 | 0.63 | 0.24 | 0.29 |
|  | 0.3 | -0.09 | 1.24 | 0.63 | 0.12 | 0.17 | 0.17 | -1.15 | -1.72 | 1.04 | 0.76 | -0.49 | 0.19 | 0.74 | 1.22 | 0.54 | 0.48 | 1.87 | 0.59 | -0.35 | 0.42 | 0.87 | 1.32 | 1.09 | 1.21 |
|  | 1 | -0.11 | 1.77 | 1.58 | 0.91 | 0.49 | 0.55 | -0.76 | -1.47 | 1.43 | 1.30 | 0.24 | 0.73 | 1.06 | 1.20 | 0.73 | 0.64 | 2.35 | 1.40 | 0.40 | 1.05 | 1.36 | 1.47 | 1.19 | 1.39 |
|  | 3 | -0.73 | 2.06 | 1.23 | 0.74 | 0.27 | -0.29 | -2.23 | -3.19 | 1.95 | 1.54 | 0.57 | 0.77 | 0.64 | 0.61 | -0.32 | -1.31 | 2.90 | 1.69 | 0.50 | 0.93 | 1.18 | 1.21 | 0.70 | 0.04 |
| NAcc | V+K | 0.63 | 1.20 | 0.60 | 0.41 | 0.72 | 0.94 | -0.07 | -0.05 | 0.64 | 0.38 | 0.29 | 0.40 | 0.50 | 0.71 | 0.21 | 0.08 | 0.77 | 0.27 | 0.20 | 0.47 | 0.80 | 0.94 | 0.46 | 0.45 |
|  | 0.3 | 0.14 | 1.39 | 0.98 | 0.43 | 0.17 | 0.71 | -0.13 | -0.54 | 0.72 | 0.68 | -0.01 | 0.78 | 1.13 | 1.76 | 1.05 | 0.89 | 1.28 | 0.11 | -0.25 | 0.53 | 1.42 | 1.82 | 1.39 | 1.37 |
|  | 1 | -0.26 | 0.82 | 0.15 | -0.09 | 0.33 | 0.58 | 0.05 | -0.18 | 1.18 | 1.08 | 0.42 | 1.15 | 1.93 | 2.14 | 1.82 | 1.66 | 2.02 | 0.87 | 0.46 | 1.28 | 2.43 | 2.35 | 1.95 | 1.83 |
|  | 3 | -1.14 | 0.27 | -0.47 | -0.21 | 0.13 | 0.66 | -0.11 | -0.78 | 0.95 | 0.32 | -0.12 | 0.21 | 0.93 | 1.18 | 0.95 | 0.20 | 1.57 | 0.43 | 0.01 | 0.69 | 2.25 | 2.01 | 1.71 | 0.95 |
| PFC | V+K | 0.61 | 1.15 | 0.73 | 0.44 | 0.43 | 0.40 | -0.40 | -0.40 | 0.66 | 0.32 | 0.21 | 0.14 | 0.20 | 0.36 | -0.13 | -0.39 | 0.68 | 0.19 | 0.05 | 0.19 | 0.55 | 0.80 | 0.27 | 0.10 |
|  | 0.3 | 0.46 | 1.30 | 0.53 | -0.06 | -0.17 | 0.72 | -0.02 | -0.16 | 0.89 | 0.80 | 0.50 | 0.96 | 1.39 | 2.01 | 1.37 | 1.27 | 1.33 | 0.29 | 0.07 | 0.57 | 1.20 | 1.79 | 1.58 | 1.45 |
|  | 1 | 0.05 | 0.83 | -0.30 | -0.70 | -0.61 | 0.13 | -0.18 | -0.38 | 0.93 | 1.04 | 0.43 | 0.65 | 1.22 | 1.79 | 1.33 | 1.36 | 1.65 | 1.02 | 0.66 | 1.02 | 1.78 | 2.08 | 1.49 | 1.65 |
|  | 3 | -0.74 | 0.38 | -1.51 | -1.73 | -0.97 | 0.01 | -0.56 | -0.79 | 1.35 | 0.58 | -0.30 | -0.38 | 0.51 | 1.20 | 1.15 | 0.63 | 1.87 | 0.60 | 0.14 | 0.37 | 1.67 | 1.81 | 1.70 | 1.12 |
| Thalamus | V+K | 0.39 | 0.99 | 0.44 | 0.15 | 0.45 | 0.53 | -0.37 | -0.53 | 0.59 | 0.22 | -0.05 | 0.05 | 0.31 | 0.50 | -0.08 | -0.30 | 0.72 | 0.07 | -0.18 | 0.19 | 0.64 | 0.77 | 0.36 | 0.36 |
|  | 0.3 | 0.07 | 1.23 | 0.68 | 0.06 | 0.03 | 0.54 | -0.31 | -0.70 | 0.75 | 0.63 | -0.26 | 0.36 | 0.90 | 1.53 | 1.09 | 1.18 | 1.35 | 0.27 | -0.16 | 0.49 | 1.02 | 1.46 | 1.41 | 1.58 |
|  | 1 | 0.69 | 1.53 | 0.27 | 0.45 | 0.52 | 0.66 | -0.46 | -0.76 | 1.13 | 0.76 | 0.15 | 0.61 | 1.15 | 1.48 | 1.27 | 1.20 | 1.91 | 0.74 | 0.42 | 0.91 | 1.50 | 1.63 | 1.41 | 1.41 |
|  | 3 | -1.25 | 0.58 | -0.66 | -0.69 | -0.54 | -0.25 | -1.31 | -2.16 | 1.29 | 0.56 | -0.15 | 0.06 | 0.27 | 0.55 | 0.22 | -0.51 | 1.93 | 0.71 | -0.08 | 0.41 | 1.26 | 1.19 | 1.00 | 0.43 |
| 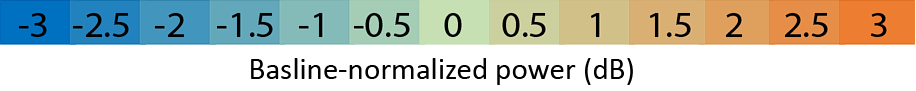 | | | | | | | | | | | | | | | | | | | | | | | | | |

Supplementary Table 3: Table of p values at 10-30 mins for group receiving clozapine. P-values were determined using mixed-model regression and consulting pairwise comparisons vs ‘vehicle + ketamine’ as described in Methods 2.2.5 and are given to 3 decimal places. *Dose is given in mg/kg; V = Vehicle; K = ketamine 10 mg/kg*

| **CLOZAPINE 10-30 MINS** | | | | | | | | | | | | | | | | | | | | | | | | | |
| --- | --- | --- | --- | --- | --- | --- | --- | --- | --- | --- | --- | --- | --- | --- | --- | --- | --- | --- | --- | --- | --- | --- | --- | --- | --- |
| Region | Dose | **ACTIVE** | | | | | | | | **ANY** | | | | | | | | **INACTIVE** | | | | | | | |
|  |  | 0-4 | 4-10 | 10-20 | 20-30 | 30-60 | 60-130 | 130-160 | 160-200 | 0-4 | 4-10 | 10-20 | 20-30 | 30-60 | 60-130 | 130-160 | 160-200 | 0-4 | 4-10 | 10-20 | 20-30 | 30-60 | 60-130 | 130-160 | 160-200 |
| AC | V+K |  |  |  |  |  |  |  |  |  |  |  |  |  |  |  |  |  |  |  |  |  |  |  |  |
|  | 0.3 | 0.582 | 0.677 | 0.646 | 0.999 | 0.865 | 0.990 | 0.527 | 0.106 | 0.537 | 0.056 | 0.318 | 0.537 | 0.096 | 0.011 | 0.107 | 0.200 | 0.008 | 0.372 | 0.894 | 0.179 | 0.260 | 0.022 | 0.006 | 0.178 |
|  | 1 | 0.705 | 0.105 | 0.006 | 0.117 | 0.690 | 0.885 | 0.970 | 0.337 | 0.031 | 0.001 | 0.348 | 0.001 | 0.003 | 0.018 | 0.030 | 0.110 | 0.001 | 0.001 | 0.005 | 0.001 | 0.003 | 0.005 | 0.003 | 0.087 |
|  | 3 | 0.001 | 0.002 | 0.040 | 0.268 | 1.000 | 0.617 | 0.003 | 0.001 | 0.001 | 0.001 | 0.025 | 0.001 | 0.239 | 0.751 | 0.980 | 0.227 | 0.001 | 0.001 | 0.002 | 0.001 | 0.027 | 0.088 | 0.298 | 0.953 |
| NAcc | V+K |  |  |  |  |  |  |  |  |  |  |  |  |  |  |  |  |  |  |  |  |  |  |  |  |
|  | 0.3 | 0.767 | 0.288 | 0.144 | 0.354 | 0.610 | 0.981 | 0.900 | 0.964 | 0.998 | 0.865 | 0.682 | 0.459 | 0.596 | 0.197 | 0.221 | 0.423 | 0.508 | 0.914 | 0.468 | 0.969 | 0.476 | 0.133 | 0.218 | 0.414 |
|  | 1 | 0.195 | 1.000 | 0.947 | 1.000 | 0.953 | 0.725 | 0.753 | 1.000 | 0.297 | 0.064 | 0.814 | 0.025 | 0.026 | 0.034 | 0.003 | 0.017 | 0.003 | 0.236 | 0.456 | 0.004 | 0.007 | 0.007 | 0.013 | 0.093 |
|  | 3 | 0.001 | 0.452 | 0.001 | 0.091 | 0.054 | 0.845 | 1.000 | 0.233 | 0.700 | 0.998 | 0.249 | 0.934 | 0.791 | 0.763 | 0.248 | 0.993 | 0.069 | 0.961 | 0.797 | 0.669 | 0.010 | 0.030 | 0.030 | 0.797 |
| PFC | V+K |  |  |  |  |  |  |  |  |  |  |  |  |  |  |  |  |  |  |  |  |  |  |  |  |
|  | 0.3 | 1.000 | 0.791 | 0.845 | 0.142 | 0.133 | 0.316 | 0.266 | 0.911 | 0.895 | 0.406 | 0.787 | 0.158 | 0.140 | 0.007 | 0.007 | 0.025 | 0.260 | 0.991 | 1.000 | 0.329 | 0.558 | 0.085 | 0.008 | 0.109 |
|  | 1 | 0.259 | 0.142 | 0.107 | 0.067 | 0.004 | 1.000 | 0.410 | 0.999 | 0.870 | 0.081 | 0.812 | 0.450 | 0.243 | 0.021 | 0.009 | 0.014 | 0.029 | 0.033 | 0.011 | 0.002 | 0.067 | 0.013 | 0.012 | 0.046 |
|  | 3 | 0.001 | 0.040 | 0.001 | 0.001 | 0.001 | 0.972 | 1.000 | 0.866 | 0.083 | 0.791 | 0.327 | 0.460 | 0.942 | 0.308 | 0.025 | 0.271 | 0.004 | 0.503 | 0.978 | 0.852 | 0.111 | 0.068 | 0.003 | 0.297 |
| Thalamus | V+K |  |  |  |  |  |  |  |  |  |  |  |  |  |  |  |  |  |  |  |  |  |  |  |  |
|  | 0.3 | 0.732 | 0.136 | 0.279 | 0.997 | 0.289 | 0.083 | 0.998 | 0.983 | 0.887 | 0.257 | 0.746 | 0.393 | 0.206 | 0.001 | 0.007 | 0.021 | 0.026 | 0.800 | 0.988 | 0.595 | 0.213 | 0.003 | 0.001 | 0.054 |
|  | 1 | 1.000 | 0.597 | 0.903 | 0.306 | 0.028 | 0.939 | 0.992 | 0.963 | 0.067 | 0.049 | 0.736 | 0.036 | 0.041 | 0.002 | 0.003 | 0.025 | 0.001 | 0.004 | 0.027 | 0.026 | 0.008 | 0.001 | 0.001 | 0.150 |
|  | 3 | 0.001 | 1.000 | 0.001 | 0.001 | 0.001 | 0.021 | 0.052 | 0.008 | 0.021 | 0.384 | 0.962 | 1.000 | 0.999 | 0.999 | 0.851 | 0.980 | 0.001 | 0.003 | 0.941 | 0.824 | 0.458 | 0.547 | 0.164 | 1.000 |

Supplementary Table 4: Table of averaged power spectra at 10-30 mins. Pre-treatment with Naltrexone was given at 0 mins. Separated by Active (left), Any (middle) and Inactive (right) epochs. Values are given in dB change from baseline. dB is a logarithmic scale, meaning that –3dB = 50% of original value, whilst 3dB = 200% of original value. Values that are significantly different vs ketamine are coloured according to the valence of change from baseline. *Dose is given in mg/kg;* *V = Vehicle; K = ketamine 10 mg/kg*

| **NALTREXONE 10-30 MINS** | | | | | | | | | | | | | | | | | | | | | | | | | |
| --- | --- | --- | --- | --- | --- | --- | --- | --- | --- | --- | --- | --- | --- | --- | --- | --- | --- | --- | --- | --- | --- | --- | --- | --- | --- |
| Region | Dose | **ACTIVE** | | | | | | | | **ANY** | | | | | | | | **INACTIVE** | | | | | | | |
|  |  | 0-4 | 4-10 | 10-20 | 20-30 | 30-60 | 60-130 | 130-160 | 160-200 | 0-4 | 4-10 | 10-20 | 20-30 | 30-60 | 60-130 | 130-160 | 160-200 | 0-4 | 4-10 | 10-20 | 20-30 | 30-60 | 60-130 | 130-160 | 160-200 |
| AC | V+K | 0.48 | 0.78 | 0.13 | -0.08 | 0.32 | 0.34 | -0.30 | -0.44 | 0.36 | -0.04 | -0.66 | -0.12 | 0.41 | 0.75 | 0.40 | 0.67 | 0.50 | -0.40 | -1.12 | -0.32 | 0.45 | 0.92 | 0.61 | 1.33 |
|  | 1 | 0.02 | 0.55 | -0.07 | -0.04 | 0.40 | 0.33 | -0.71 | -0.82 | 0.01 | -0.25 | -0.07 | 0.28 | 0.19 | -0.05 | -0.57 | -1.25 | 0.08 | -0.62 | -0.65 | 0.11 | 0.52 | 0.54 | 0.27 | -0.10 |
|  | 3 | -0.77 | 0.55 | -0.27 | -0.14 | 0.30 | 0.00 | -0.88 | -1.05 | -0.14 | -0.20 | -0.26 | 0.08 | -0.06 | -0.66 | -1.18 | -1.77 | -0.02 | -0.66 | -0.94 | -0.12 | 0.23 | -0.04 | -0.29 | -0.47 |
|  | 10 | -0.71 | 0.24 | -0.35 | -0.06 | 0.29 | -0.64 | -1.04 | -0.87 | -0.27 | -0.37 | -0.08 | -0.03 | -0.42 | -1.89 | -2.29 | -2.64 | -0.40 | -0.70 | -0.76 | -0.27 | -0.11 | -1.42 | -1.83 | -1.78 |
| NAcc | V+K | 0.45 | 0.58 | 0.25 | -0.16 | 0.29 | 0.67 | -0.21 | -0.13 | 0.34 | 0.36 | -0.03 | 0.32 | 0.78 | 1.07 | 0.43 | 0.83 | 0.50 | -0.06 | -0.34 | 0.27 | 0.97 | 1.21 | 0.39 | 1.20 |
|  | 1 | 0.08 | 0.52 | 0.35 | 0.63 | 0.88 | 0.67 | -0.29 | -0.49 | -0.13 | -0.77 | -0.45 | -0.14 | -0.11 | -0.03 | -0.12 | -0.76 | -0.18 | -1.17 | -0.99 | -0.37 | 0.27 | 0.51 | 0.39 | -0.01 |
|  | 3 | -0.57 | 0.73 | -0.13 | 0.14 | 0.53 | 0.17 | -0.78 | -0.89 | -0.17 | -0.75 | -0.64 | -0.42 | -0.30 | -0.83 | -0.98 | -1.43 | -0.15 | -1.11 | -1.08 | -0.37 | 0.24 | -0.11 | -0.18 | -0.36 |
|  | 10 | -0.70 | -0.03 | -0.24 | 0.13 | 0.39 | -0.89 | -1.26 | -1.15 | -0.24 | -0.71 | -0.12 | -0.26 | -0.58 | -2.49 | -2.45 | -2.67 | -0.34 | -0.63 | -0.38 | -0.06 | 0.13 | -1.83 | -2.01 | -1.67 |
| PFC | V+K | 0.47 | 0.90 | 0.48 | 0.17 | 0.32 | 0.38 | -0.23 | 0.04 | -0.09 | 0.08 | 0.11 | 0.48 | 0.70 | 0.88 | 0.29 | 0.58 | 0.10 | -0.36 | -0.41 | 0.18 | 0.96 | 1.10 | 0.33 | 1.04 |
|  | 1 | -0.11 | 0.04 | -0.51 | -0.83 | -0.18 | 0.28 | -0.66 | -0.57 | -0.31 | -0.96 | -0.75 | -0.88 | -0.84 | -0.38 | -0.63 | -1.14 | -0.17 | -0.92 | -0.73 | -0.39 | -0.01 | 0.45 | 0.32 | -0.02 |
|  | 3 | -0.46 | -0.42 | -1.48 | -1.60 | -0.86 | -0.58 | -1.22 | -1.14 | -0.30 | -1.00 | -1.26 | -1.37 | -1.26 | -1.34 | -1.56 | -1.83 | -0.10 | -0.94 | -1.13 | -0.62 | -0.23 | -0.39 | -0.55 | -0.59 |
|  | 10 | -0.63 | -0.76 | -1.32 | -1.67 | -0.83 | -1.06 | -1.55 | -1.51 | -0.48 | -1.29 | -1.11 | -1.43 | -1.39 | -2.40 | -2.54 | -2.58 | -0.38 | -0.73 | -0.58 | -0.35 | -0.16 | -1.59 | -1.97 | -1.51 |
| Thalamus | V+K | 0.50 | 0.50 | 0.29 | 0.05 | 0.40 | 0.53 | -0.15 | -0.24 | 0.24 | 0.26 | -0.08 | 0.37 | 0.60 | 0.81 | 0.44 | 0.78 | 0.39 | -0.27 | -0.55 | 0.08 | 0.55 | 0.82 | 0.39 | 1.14 |
|  | 1 | 0.27 | 0.84 | 0.44 | 0.21 | 0.56 | 0.35 | -0.57 | -0.70 | -0.16 | -0.43 | -0.07 | 0.11 | 0.02 | 0.02 | -0.12 | -0.67 | -0.09 | -0.60 | -0.46 | 0.03 | 0.25 | 0.41 | 0.31 | -0.09 |
|  | 3 | -0.49 | 0.50 | -0.49 | -0.46 | -0.02 | -0.09 | -1.10 | -0.97 | -0.28 | -0.60 | -0.55 | -0.48 | -0.60 | -0.87 | -1.13 | -1.49 | -0.15 | -0.88 | -0.93 | -0.33 | -0.06 | -0.20 | -0.27 | -0.36 |
|  | 10 | -0.65 | -0.07 | -0.26 | -0.01 | 0.32 | -0.68 | -0.99 | -0.88 | -0.48 | -0.81 | -0.34 | -0.21 | -0.54 | -2.01 | -2.10 | -2.33 | -0.45 | -0.89 | -0.81 | -0.27 | -0.11 | -1.48 | -1.64 | -1.39 |
| 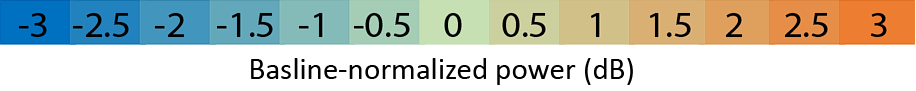 | | | | | | | | | | | | | | | | | | | | | | | | | |

Supplementary Table 5: Table of p values at 10-30 mins for group receiving naltrexone. P-values were determined using mixed-model regression and consulting pairwise comparisons vs ‘vehicle + ketamine’ as described in Methods 2.2.5 and are given to 3 decimal places. *Dose is given in mg/kg; V = Vehicle; K = ketamine 10 mg/kg*

| **NALTREXONE 10-30 MINS** | | | | | | | | | | | | | | | | | | | | | | | | | |
| --- | --- | --- | --- | --- | --- | --- | --- | --- | --- | --- | --- | --- | --- | --- | --- | --- | --- | --- | --- | --- | --- | --- | --- | --- | --- |
| Region | Dose | **ACTIVE** | | | | | | | | **ANY** | | | | | | | | **INACTIVE** | | | | | | | |
|  |  | 0-4 | 4-10 | 10-20 | 20-30 | 30-60 | 60-130 | 130-160 | 160-200 | 0-4 | 4-10 | 10-20 | 20-30 | 30-60 | 60-130 | 130-160 | 160-200 | 0-4 | 4-10 | 10-20 | 20-30 | 30-60 | 60-130 | 130-160 | 160-200 |
| AC | V+K |  |  |  |  |  |  |  |  |  |  |  |  |  |  |  |  |  |  |  |  |  |  |  |  |
|  | 1 | 0.427 | 0.912 | 0.770 | 0.823 | 0.965 | 1.000 | 0.682 | 0.837 | 0.506 | 0.898 | 0.458 | 0.446 | 0.772 | 0.077 | 0.035 | 0.002 | 0.568 | 0.917 | 0.708 | 0.598 | 1.000 | 0.635 | 0.675 | 0.007 |
|  | 3 | 0.001 | 0.923 | 0.235 | 1.000 | 0.999 | 0.256 | 0.215 | 0.492 | 0.193 | 0.966 | 0.737 | 0.633 | 0.244 | 0.001 | 0.001 | 0.001 | 0.449 | 0.867 | 0.987 | 0.869 | 0.577 | 0.020 | 0.022 | 0.001 |
|  | 10 | 0.002 | 0.441 | 0.108 | 0.962 | 0.999 | 0.001 | 0.130 | 0.798 | 0.061 | 0.794 | 0.383 | 0.995 | 0.009 | 0.001 | 0.001 | 0.001 | 0.029 | 0.849 | 0.780 | 0.988 | 0.045 | 0.001 | 0.001 | 0.001 |
| NAcc | V+K |  |  |  |  |  |  |  |  |  |  |  |  |  |  |  |  |  |  |  |  |  |  |  |  |
|  | 1 | 0.862 | 0.998 | 0.999 | 0.010 | 0.202 | 1.000 | 1.000 | 0.772 | 0.566 | 0.003 | 0.345 | 0.034 | 0.061 | 0.073 | 0.367 | 0.010 | 0.440 | 0.032 | 0.238 | 0.001 | 0.084 | 0.383 | 1.000 | 0.020 |
|  | 3 | 0.146 | 0.972 | 0.407 | 0.529 | 0.843 | 0.113 | 0.114 | 0.176 | 0.475 | 0.003 | 0.158 | 0.003 | 0.026 | 0.001 | 0.003 | 0.001 | 0.452 | 0.033 | 0.143 | 0.001 | 0.072 | 0.017 | 0.297 | 0.002 |
|  | 10 | 0.087 | 0.042 | 0.143 | 0.855 | 0.988 | 0.001 | 0.001 | 0.040 | 0.379 | 0.006 | 0.857 | 0.002 | 0.003 | 0.001 | 0.001 | 0.001 | 0.221 | 0.650 | 0.998 | 0.015 | 0.029 | 0.001 | 0.001 | 0.001 |
| PFC | V+K |  |  |  |  |  |  |  |  |  |  |  |  |  |  |  |  |  |  |  |  |  |  |  |  |
|  | 1 | 0.651 | 0.673 | 0.650 | 0.468 | 0.934 | 0.998 | 0.889 | 0.810 | 0.938 | 0.004 | 0.020 | 0.002 | 0.005 | 0.087 | 0.245 | 0.043 | 0.969 | 0.542 | 0.781 | 0.118 | 0.003 | 0.373 | 0.995 | 0.112 |
|  | 3 | 0.223 | 0.272 | 0.084 | 0.062 | 0.461 | 0.257 | 0.312 | 0.275 | 0.934 | 0.001 | 0.001 | 0.001 | 0.001 | 0.001 | 0.002 | 0.002 | 0.993 | 0.437 | 0.090 | 0.007 | 0.001 | 0.004 | 0.092 | 0.004 |
|  | 10 | 0.127 | 0.128 | 0.148 | 0.074 | 0.512 | 0.044 | 0.124 | 0.108 | 0.698 | 0.001 | 0.001 | 0.001 | 0.001 | 0.001 | 0.001 | 0.001 | 0.686 | 0.813 | 0.973 | 0.141 | 0.001 | 0.001 | 0.001 | 0.001 |
| Thalamus | V+K |  |  |  |  |  |  |  |  |  |  |  |  |  |  |  |  |  |  |  |  |  |  |  |  |
|  | 1 | 0.958 | 0.277 | 0.896 | 0.472 | 0.877 | 0.899 | 0.508 | 0.667 | 0.698 | 0.039 | 0.974 | 0.707 | 0.240 | 0.142 | 0.262 | 0.009 | 0.685 | 0.514 | 0.952 | 0.342 | 0.418 | 0.661 | 0.996 | 0.006 |
|  | 3 | 0.120 | 0.999 | 0.017 | 0.257 | 0.326 | 0.043 | 0.006 | 0.306 | 0.553 | 0.016 | 0.440 | 0.006 | 0.002 | 0.001 | 0.001 | 0.001 | 0.880 | 0.253 | 0.441 | 0.016 | 0.014 | 0.016 | 0.189 | 0.001 |
|  | 10 | 0.043 | 0.383 | 0.117 | 0.998 | 0.983 | 0.001 | 0.005 | 0.295 | 0.154 | 0.001 | 0.693 | 0.064 | 0.002 | 0.001 | 0.001 | 0.001 | 0.077 | 0.227 | 0.718 | 0.009 | 0.006 | 0.001 | 0.001 | 0.001 |

Supplementary Table 6: Table of averaged power spectra at 40-70 mins. Pre-treatment with Clozapine was given at 0 mins. Separated by Active (left), Any (middle) and Inactive (right) epochs. Values are given in dB change from baseline. dB is a logarithmic scale, meaning that –3dB = 50% of original value, whilst 3dB = 200% of original value. Values that are significantly different vs ketamine are coloured according to the valence of change from baseline. *Dose is given in mg/kg;* *V = Vehicle; K = ketamine 10 mg/kg*

| **CLOZAPINE 40-70 MINS** | | | | | | | | | | | | | | | | | | | | | | | | | |
| --- | --- | --- | --- | --- | --- | --- | --- | --- | --- | --- | --- | --- | --- | --- | --- | --- | --- | --- | --- | --- | --- | --- | --- | --- | --- |
| Region | Dose | **ACTIVE** | | | | | | | | **ANY** | | | | | | | | **INACTIVE** | | | | | | | |
|  |  | 0-4 | 4-10 | 10-20 | 20-30 | 30-60 | 60-130 | 130-160 | 160-200 | 0-4 | 4-10 | 10-20 | 20-30 | 30-60 | 60-130 | 130-160 | 160-200 | 0-4 | 4-10 | 10-20 | 20-30 | 30-60 | 60-130 | 130-160 | 160-200 |
| AC | V+K | -0.86 | 1.27 | -0.24 | -0.43 | 0.99 | 0.47 | 0.06 | -1.94 | 0.21 | -1.30 | -3.05 | -1.26 | 1.08 | 0.69 | 1.04 | -1.44 | 0.49 | -1.84 | -3.96 | -1.52 | 1.70 | 1.58 | 2.33 | 0.41 |
|  | 0.3 | -0.83 | 1.01 | -0.86 | -0.65 | 1.39 | 0.42 | 0.79 | -3.26 | 0.85 | -0.16 | -2.92 | -0.41 | 2.42 | 1.95 | 3.09 | -0.81 | 1.50 | -0.30 | -3.39 | -0.44 | 3.10 | 2.88 | 4.45 | 1.27 |
|  | 1 | -1.26 | 1.92 | 0.45 | 0.54 | 2.07 | 0.86 | 1.95 | -3.11 | 0.82 | 0.80 | -1.84 | 0.12 | 2.49 | 1.80 | 3.60 | -0.88 | 1.45 | 0.89 | -2.16 | 0.13 | 2.97 | 2.65 | 4.73 | 0.90 |
|  | 3 | -1.74 | 1.50 | -0.13 | 0.59 | 1.94 | 0.09 | 1.17 | -4.49 | 0.94 | 1.31 | -0.41 | 1.18 | 2.60 | 1.94 | 3.42 | -2.46 | 1.91 | 1.53 | -0.75 | 1.20 | 3.19 | 2.95 | 4.66 | -0.62 |
| NAcc | V+K | -0.23 | 1.21 | -0.45 | -0.51 | 0.54 | 1.16 | 3.05 | 0.60 | 0.03 | -1.19 | -2.03 | -1.15 | 0.45 | 0.87 | 3.69 | 0.32 | 0.02 | -1.48 | -2.37 | -0.82 | 1.63 | 1.77 | 4.63 | 1.31 |
|  | 0.3 | -0.15 | 0.64 | -1.28 | -1.54 | -0.49 | 0.31 | 4.47 | -0.92 | 0.62 | -0.15 | -2.67 | -0.73 | 1.30 | 2.01 | 6.14 | 0.35 | 0.91 | -0.29 | -3.02 | -0.42 | 2.92 | 3.33 | 7.37 | 1.90 |
|  | 1 | -0.77 | 0.66 | -1.82 | -1.66 | -0.53 | 0.25 | 4.61 | -0.56 | 0.98 | 0.47 | -2.06 | -0.62 | 1.43 | 2.06 | 6.71 | 0.94 | 1.34 | 0.63 | -2.15 | -0.16 | 2.73 | 3.25 | 7.76 | 2.11 |
|  | 3 | -1.33 | 0.34 | -1.77 | -1.44 | -0.58 | 0.65 | 5.97 | -1.79 | 0.26 | 0.33 | -1.70 | -0.54 | 1.08 | 2.52 | 7.50 | -0.36 | 0.75 | 0.56 | -1.73 | 0.10 | 2.78 | 3.90 | 8.51 | 0.93 |
| PFC | V+K | -0.63 | 0.07 | -1.64 | -2.06 | -0.51 | 0.04 | 1.65 | -0.40 | -0.09 | -1.55 | -2.76 | -1.97 | 0.22 | 0.40 | 2.77 | -0.17 | -0.05 | -1.68 | -2.81 | -1.15 | 1.82 | 1.79 | 3.95 | 1.04 |
|  | 0.3 | 0.01 | 0.37 | -2.54 | -2.59 | -0.75 | 0.15 | 3.81 | -0.63 | 0.39 | -0.46 | -2.35 | -0.79 | 1.73 | 2.22 | 6.01 | 1.10 | 0.65 | -0.57 | -2.29 | -0.14 | 3.22 | 3.36 | 7.04 | 2.27 |
|  | 1 | -1.05 | 0.54 | -2.42 | -2.43 | -0.63 | -0.49 | 4.83 | -0.61 | 0.62 | 0.53 | -1.87 | -0.88 | 1.40 | 1.49 | 6.64 | 1.05 | 1.06 | 0.81 | -1.43 | 0.07 | 2.76 | 2.62 | 7.39 | 1.97 |
|  | 3 | -1.19 | 0.45 | -2.83 | -2.51 | -0.11 | 0.05 | 5.02 | -2.47 | 0.65 | 0.70 | -1.67 | -0.70 | 2.04 | 2.47 | 7.21 | -0.55 | 1.10 | 0.90 | -1.32 | 0.19 | 3.58 | 3.72 | 8.05 | 0.48 |
| Thalamus | V+K | -0.69 | 1.06 | -0.68 | -0.93 | 0.38 | 0.89 | 1.42 | -0.58 | -0.11 | -1.32 | -2.87 | -1.59 | 0.41 | 0.89 | 2.14 | -0.42 | 0.04 | -1.83 | -3.51 | -1.49 | 1.34 | 1.68 | 3.21 | 0.97 |
|  | 0.3 | -0.19 | 1.12 | -1.01 | -1.38 | 0.10 | 0.89 | 3.08 | -0.78 | 0.37 | -0.16 | -2.69 | -0.82 | 1.51 | 2.27 | 4.77 | 0.96 | 0.73 | -0.41 | -3.02 | -0.68 | 2.38 | 2.99 | 5.57 | 2.14 |
|  | 1 | -0.36 | 1.58 | -1.02 | -0.78 | 0.26 | 0.73 | 3.28 | -1.35 | 0.58 | 0.47 | -2.01 | -0.51 | 1.36 | 2.04 | 5.32 | 0.44 | 1.04 | 0.52 | -2.12 | -0.25 | 2.16 | 2.80 | 6.10 | 1.50 |
|  | 3 | -0.93 | 0.99 | -1.52 | -1.44 | -0.45 | -0.03 | 3.35 | -3.05 | 0.76 | 0.56 | -1.86 | -0.64 | 0.80 | 1.65 | 5.11 | -1.55 | 1.34 | 0.74 | -2.01 | -0.24 | 2.08 | 2.74 | 6.18 | -0.11 |
| 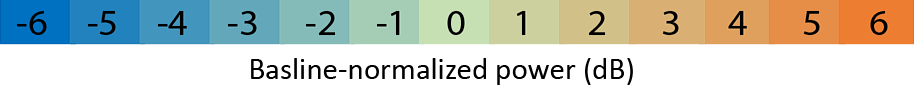 | | | | | | | | | | | | | | | | | | | | | | | | | |

Supplementary Table 7: Table of p values at 40-70 mins for group receiving clozapine. P-values were determined using mixed-model regression and consulting pairwise comparisons vs ‘vehicle + ketamine’ as described in Methods 2.2.5 and are given to 3 decimal places. *Dose is given in mg/kg; V = Vehicle; K = ketamine 10 mg/kg*

| **CLOZAPINE 40-70 MINS** | | | | | | | | | | | | | | | | | | | | | | | | | |
| --- | --- | --- | --- | --- | --- | --- | --- | --- | --- | --- | --- | --- | --- | --- | --- | --- | --- | --- | --- | --- | --- | --- | --- | --- | --- |
| Region | Dose | **ACTIVE** | | | | | | | | **ANY** | | | | | | | | **INACTIVE** | | | | | | | |
|  |  | 0-4 | 4-10 | 10-20 | 20-30 | 30-60 | 60-130 | 130-160 | 160-200 | 0-4 | 4-10 | 10-20 | 20-30 | 30-60 | 60-130 | 130-160 | 160-200 | 0-4 | 4-10 | 10-20 | 20-30 | 30-60 | 60-130 | 130-160 | 160-200 |
| AC | V+K |  |  |  |  |  |  |  |  |  |  |  |  |  |  |  |  |  |  |  |  |  |  |  |  |
|  | 0.3 | 0.999 | 0.700 | 0.696 | 0.941 | 0.275 | 0.998 | 0.238 | 0.008 | 0.679 | 0.001 | 0.299 | 0.001 | 0.001 | 0.001 | 0.001 | 0.044 | 0.627 | 0.001 | 0.002 | 0.001 | 0.001 | 0.001 | 0.001 | 0.010 |
|  | 1 | 0.652 | 0.232 | 0.629 | 0.073 | 0.001 | 0.589 | 0.001 | 0.109 | 0.592 | 0.001 | 0.001 | 0.001 | 0.001 | 0.001 | 0.001 | 0.003 | 0.835 | 0.001 | 0.001 | 0.001 | 0.001 | 0.001 | 0.001 | 0.031 |
|  | 3 | 0.033 | 0.775 | 0.998 | 0.050 | 0.001 | 0.915 | 0.016 | 0.001 | 0.606 | 0.001 | 0.001 | 0.001 | 0.001 | 0.001 | 0.001 | 0.180 | 0.032 | 0.001 | 0.001 | 0.001 | 0.001 | 0.001 | 0.001 | 0.008 |
| NAcc | V+K |  |  |  |  |  |  |  |  |  |  |  |  |  |  |  |  |  |  |  |  |  |  |  |  |
|  | 0.3 | 0.999 | 0.585 | 0.779 | 0.725 | 0.813 | 0.788 | 0.004 | 0.152 | 0.622 | 0.004 | 0.536 | 0.019 | 0.018 | 0.107 | 0.001 | 0.903 | 0.552 | 0.005 | 0.438 | 0.047 | 0.002 | 0.003 | 0.001 | 0.422 |
|  | 1 | 0.802 | 0.317 | 0.130 | 0.997 | 0.825 | 0.518 | 0.006 | 0.143 | 0.076 | 0.001 | 0.996 | 0.001 | 0.001 | 0.069 | 0.001 | 0.540 | 0.020 | 0.001 | 0.867 | 0.001 | 0.003 | 0.004 | 0.001 | 0.542 |
|  | 3 | 0.186 | 0.649 | 0.005 | 0.448 | 0.078 | 0.629 | 0.001 | 0.001 | 0.709 | 0.001 | 0.610 | 0.001 | 0.026 | 0.006 | 0.001 | 0.330 | 0.102 | 0.001 | 0.196 | 0.001 | 0.002 | 0.001 | 0.001 | 0.570 |
| PFC | V+K |  |  |  |  |  |  |  |  |  |  |  |  |  |  |  |  |  |  |  |  |  |  |  |  |
|  | 0.3 | 0.447 | 0.958 | 0.200 | 0.997 | 0.543 | 0.603 | 0.001 | 0.990 | 1.000 | 0.015 | 0.361 | 0.001 | 0.001 | 0.001 | 0.001 | 0.017 | 0.981 | 0.014 | 0.268 | 0.001 | 0.001 | 0.002 | 0.001 | 0.003 |
|  | 1 | 0.802 | 0.723 | 0.805 | 0.017 | 0.025 | 0.969 | 0.001 | 0.980 | 0.453 | 0.001 | 0.007 | 0.001 | 0.001 | 0.020 | 0.001 | 0.022 | 0.367 | 0.001 | 0.001 | 0.001 | 0.009 | 0.080 | 0.001 | 0.019 |
|  | 3 | 0.665 | 0.028 | 0.015 | 0.866 | 0.002 | 0.572 | 0.001 | 0.004 | 0.149 | 0.001 | 0.001 | 0.001 | 0.001 | 0.001 | 0.001 | 0.609 | 0.023 | 0.001 | 0.001 | 0.001 | 0.001 | 0.001 | 0.001 | 0.131 |
| Thalamus | V+K |  |  |  |  |  |  |  |  |  |  |  |  |  |  |  |  |  |  |  |  |  |  |  |  |
|  | 0.3 | 0.868 | 0.999 | 0.888 | 0.683 | 0.964 | 1.000 | 0.001 | 0.936 | 0.073 | 0.002 | 0.862 | 0.069 | 0.001 | 0.001 | 0.001 | 0.001 | 0.063 | 0.001 | 0.294 | 0.058 | 0.001 | 0.001 | 0.001 | 0.001 |
|  | 1 | 0.944 | 0.784 | 0.922 | 0.962 | 0.890 | 1.000 | 0.001 | 0.748 | 0.013 | 0.001 | 0.030 | 0.005 | 0.001 | 0.001 | 0.001 | 0.006 | 0.009 | 0.001 | 0.001 | 0.002 | 0.001 | 0.001 | 0.001 | 0.157 |
|  | 3 | 0.966 | 0.999 | 0.415 | 0.715 | 0.336 | 0.024 | 0.001 | 0.001 | 0.005 | 0.001 | 0.003 | 0.019 | 0.222 | 0.004 | 0.001 | 0.016 | 0.001 | 0.001 | 0.001 | 0.003 | 0.064 | 0.001 | 0.001 | 0.001 |

Supplementary Table 8: Table of averaged power spectra at 10-30 mins. Pre-treatment with Naltrexone was given at 0 mins. Separated by Active (left), Any (middle) and Inactive (right) epochs. Values are given in dB change from baseline. dB is a logarithmic scale, meaning that –3dB = 50% of original value, whilst 3dB = 200% of original value. Values that are significantly different vs ketamine are coloured according to the valence of change from baseline. *Dose is given in mg/kg;* *V = Vehicle; K = ketamine 10 mg/kg*

| **NALTREXONE 40-70 MINS** | | | | | | | | | | | | | | | | | | | | | | | | | |
| --- | --- | --- | --- | --- | --- | --- | --- | --- | --- | --- | --- | --- | --- | --- | --- | --- | --- | --- | --- | --- | --- | --- | --- | --- | --- |
| Region | Dose | **ACTIVE** | | | | | | | | **ANY** | | | | | | | | **INACTIVE** | | | | | | | |
|  |  | 0-4 | 4-10 | 10-20 | 20-30 | 30-60 | 60-130 | 130-160 | 160-200 | 0-4 | 4-10 | 10-20 | 20-30 | 30-60 | 60-130 | 130-160 | 160-200 | 0-4 | 4-10 | 10-20 | 20-30 | 30-60 | 60-130 | 130-160 | 160-200 |
| AC | V+K | 0.48 | 0.78 | 0.13 | -0.08 | 0.32 | 0.34 | -0.30 | -0.44 | 0.36 | -0.04 | -0.66 | -0.12 | 0.41 | 0.75 | 0.40 | 0.67 | 0.50 | -0.40 | -1.12 | -0.32 | 0.45 | 0.92 | 0.61 | 1.33 |
|  | 1 | 0.02 | 0.55 | -0.07 | -0.04 | 0.40 | 0.33 | -0.71 | -0.82 | 0.01 | -0.25 | -0.07 | 0.28 | 0.19 | -0.05 | -0.57 | -1.25 | 0.08 | -0.62 | -0.65 | 0.11 | 0.52 | 0.54 | 0.27 | -0.10 |
|  | 3 | -0.77 | 0.55 | -0.27 | -0.14 | 0.30 | 0.00 | -0.88 | -1.05 | -0.14 | -0.20 | -0.26 | 0.08 | -0.06 | -0.66 | -1.18 | -1.77 | -0.02 | -0.66 | -0.94 | -0.12 | 0.23 | -0.04 | -0.29 | -0.47 |
|  | 10 | -0.71 | 0.24 | -0.35 | -0.06 | 0.29 | -0.64 | -1.04 | -0.87 | -0.27 | -0.37 | -0.08 | -0.03 | -0.42 | -1.89 | -2.29 | -2.64 | -0.40 | -0.70 | -0.76 | -0.27 | -0.11 | -1.42 | -1.83 | -1.78 |
| NAcc | V+K | 0.45 | 0.58 | 0.25 | -0.16 | 0.29 | 0.67 | -0.21 | -0.13 | 0.34 | 0.36 | -0.03 | 0.32 | 0.78 | 1.07 | 0.43 | 0.83 | 0.50 | -0.06 | -0.34 | 0.27 | 0.97 | 1.21 | 0.39 | 1.20 |
|  | 1 | 0.08 | 0.52 | 0.35 | 0.63 | 0.88 | 0.67 | -0.29 | -0.49 | -0.13 | -0.77 | -0.45 | -0.14 | -0.11 | -0.03 | -0.12 | -0.76 | -0.18 | -1.17 | -0.99 | -0.37 | 0.27 | 0.51 | 0.39 | -0.01 |
|  | 3 | -0.57 | 0.73 | -0.13 | 0.14 | 0.53 | 0.17 | -0.78 | -0.89 | -0.17 | -0.75 | -0.64 | -0.42 | -0.30 | -0.83 | -0.98 | -1.43 | -0.15 | -1.11 | -1.08 | -0.37 | 0.24 | -0.11 | -0.18 | -0.36 |
|  | 10 | -0.70 | -0.03 | -0.24 | 0.13 | 0.39 | -0.89 | -1.26 | -1.15 | -0.24 | -0.71 | -0.12 | -0.26 | -0.58 | -2.49 | -2.45 | -2.67 | -0.34 | -0.63 | -0.38 | -0.06 | 0.13 | -1.83 | -2.01 | -1.67 |
| PFC | V+K | 0.47 | 0.90 | 0.48 | 0.17 | 0.32 | 0.38 | -0.23 | 0.04 | -0.09 | 0.08 | 0.11 | 0.48 | 0.70 | 0.88 | 0.29 | 0.58 | 0.10 | -0.36 | -0.41 | 0.18 | 0.96 | 1.10 | 0.33 | 1.04 |
|  | 1 | -0.11 | 0.04 | -0.51 | -0.83 | -0.18 | 0.28 | -0.66 | -0.57 | -0.31 | -0.96 | -0.75 | -0.88 | -0.84 | -0.38 | -0.63 | -1.14 | -0.17 | -0.92 | -0.73 | -0.39 | -0.01 | 0.45 | 0.32 | -0.02 |
|  | 3 | -0.46 | -0.42 | -1.48 | -1.60 | -0.86 | -0.58 | -1.22 | -1.14 | -0.30 | -1.00 | -1.26 | -1.37 | -1.26 | -1.34 | -1.56 | -1.83 | -0.10 | -0.94 | -1.13 | -0.62 | -0.23 | -0.39 | -0.55 | -0.59 |
|  | 10 | -0.63 | -0.76 | -1.32 | -1.67 | -0.83 | -1.06 | -1.55 | -1.51 | -0.48 | -1.29 | -1.11 | -1.43 | -1.39 | -2.40 | -2.54 | -2.58 | -0.38 | -0.73 | -0.58 | -0.35 | -0.16 | -1.59 | -1.97 | -1.51 |
| Thalamus | V+K | 0.50 | 0.50 | 0.29 | 0.05 | 0.40 | 0.53 | -0.15 | -0.24 | 0.24 | 0.26 | -0.08 | 0.37 | 0.60 | 0.81 | 0.44 | 0.78 | 0.39 | -0.27 | -0.55 | 0.08 | 0.55 | 0.82 | 0.39 | 1.14 |
|  | 1 | 0.27 | 0.84 | 0.44 | 0.21 | 0.56 | 0.35 | -0.57 | -0.70 | -0.16 | -0.43 | -0.07 | 0.11 | 0.02 | 0.02 | -0.12 | -0.67 | -0.09 | -0.60 | -0.46 | 0.03 | 0.25 | 0.41 | 0.31 | -0.09 |
|  | 3 | -0.49 | 0.50 | -0.49 | -0.46 | -0.02 | -0.09 | -1.10 | -0.97 | -0.28 | -0.60 | -0.55 | -0.48 | -0.60 | -0.87 | -1.13 | -1.49 | -0.15 | -0.88 | -0.93 | -0.33 | -0.06 | -0.20 | -0.27 | -0.36 |
|  | 10 | -0.65 | -0.07 | -0.26 | -0.01 | 0.32 | -0.68 | -0.99 | -0.88 | -0.48 | -0.81 | -0.34 | -0.21 | -0.54 | -2.01 | -2.10 | -2.33 | -0.45 | -0.89 | -0.81 | -0.27 | -0.11 | -1.48 | -1.64 | -1.39 |
| 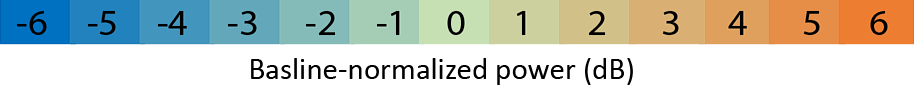 | | | | | | | | | | | | | | | | | | | | | | | | | |

Supplementary Table 9: Table of p values at 40-70 mins for group receiving naltrexone. P-values were determined using mixed-model regression and consulting pairwise comparisons vs ‘vehicle + ketamine’ as described in Methods 2.2.5 and are given to 3 decimal places and are given to 3 decimal places. *Dose is given in mg/kg; V = Vehicle; K = ketamine 10 mg/kg*

| **NALTREXONE 40-70 MINS** | | | | | | | | | | | | | | | | | | | | | | | | | |
| --- | --- | --- | --- | --- | --- | --- | --- | --- | --- | --- | --- | --- | --- | --- | --- | --- | --- | --- | --- | --- | --- | --- | --- | --- | --- |
| Region | Dose | **ACTIVE** | | | | | | | | **ANY** | | | | | | | | **INACTIVE** | | | | | | | |
|  |  | 0-4 | 4-10 | 10-20 | 20-30 | 30-60 | 60-130 | 130-160 | 160-200 | 0-4 | 4-10 | 10-20 | 20-30 | 30-60 | 60-130 | 130-160 | 160-200 | 0-4 | 4-10 | 10-20 | 20-30 | 30-60 | 60-130 | 130-160 | 160-200 |
| AC | V+K |  |  |  |  |  |  |  |  |  |  |  |  |  |  |  |  |  |  |  |  |  |  |  |  |
|  | 1 | 0.999 | 0.363 | 0.593 | 0.978 | 0.949 | 0.858 | 0.731 | 0.459 | 0.211 | 0.865 | 0.774 | 0.876 | 0.996 | 0.205 | 0.983 | 0.282 | 0.314 | 0.965 | 0.418 | 0.949 | 0.586 | 0.158 | 0.168 | 0.976 |
|  | 3 | 0.468 | 0.562 | 1.000 | 0.311 | 0.831 | 0.908 | 0.318 | 0.023 | 0.140 | 0.367 | 0.912 | 0.915 | 0.973 | 0.049 | 0.132 | 0.118 | 0.355 | 0.958 | 0.782 | 0.002 | 0.716 | 0.767 | 0.747 | 0.989 |
|  | 10 | 0.319 | 0.999 | 0.973 | 0.667 | 0.015 | 0.010 | 0.001 | 0.004 | 0.038 | 0.034 | 0.772 | 0.933 | 0.455 | 0.001 | 0.486 | 0.751 | 0.952 | 0.654 | 0.994 | 0.905 | 0.435 | 0.032 | 0.001 | 0.170 |
| NAcc | V+K |  |  |  |  |  |  |  |  |  |  |  |  |  |  |  |  |  |  |  |  |  |  |  |  |
|  | 1 | 0.942 | 0.241 | 0.040 | 0.435 | 0.509 | 0.989 | 0.729 | 0.040 | 0.281 | 0.452 | 0.506 | 1.000 | 0.209 | 0.937 | 0.568 | 0.009 | 0.012 | 0.944 | 0.652 | 0.672 | 0.968 | 0.924 | 0.994 | 0.444 |
|  | 3 | 0.863 | 0.111 | 0.051 | 0.065 | 0.208 | 0.696 | 0.387 | 0.006 | 0.078 | 0.038 | 0.220 | 0.718 | 0.250 | 0.428 | 0.385 | 0.031 | 0.089 | 0.191 | 0.986 | 0.500 | 0.994 | 1.000 | 1.000 | 0.391 |
|  | 10 | 0.445 | 0.806 | 0.298 | 0.001 | 0.008 | 0.014 | 0.001 | 0.001 | 0.025 | 0.003 | 0.190 | 0.335 | 0.028 | 0.009 | 0.492 | 0.152 | 0.913 | 0.911 | 0.996 | 0.951 | 0.466 | 0.014 | 0.001 | 0.003 |
| PFC | V+K |  |  |  |  |  |  |  |  |  |  |  |  |  |  |  |  |  |  |  |  |  |  |  |  |
|  | 1 | 0.434 | 0.157 | 0.238 | 0.575 | 0.799 | 0.995 | 0.429 | 0.344 | 0.441 | 0.966 | 0.988 | 0.992 | 1.000 | 0.982 | 0.933 | 0.767 | 0.901 | 0.706 | 0.719 | 0.735 | 0.125 | 0.988 | 0.862 | 0.164 |
|  | 3 | 0.960 | 0.088 | 0.054 | 0.026 | 0.036 | 0.229 | 0.105 | 0.109 | 0.144 | 0.192 | 0.781 | 0.828 | 0.913 | 0.511 | 0.390 | 0.222 | 0.308 | 0.714 | 0.427 | 0.133 | 0.001 | 0.610 | 0.210 | 0.223 |
|  | 10 | 0.157 | 0.344 | 0.185 | 0.039 | 0.035 | 0.031 | 0.011 | 0.088 | 0.128 | 0.340 | 0.831 | 0.903 | 1.000 | 0.846 | 0.991 | 0.985 | 1.000 | 0.969 | 1.000 | 0.652 | 0.007 | 0.001 | 0.001 | 0.008 |
| Thalamus | V+K |  |  |  |  |  |  |  |  |  |  |  |  |  |  |  |  |  |  |  |  |  |  |  |  |
|  | 1 | 0.072 | 0.331 | 0.004 | 0.084 | 0.882 | 0.890 | 0.588 | 0.373 | 0.080 | 1.000 | 0.990 | 1.000 | 0.748 | 0.949 | 1.000 | 0.008 | 0.450 | 0.978 | 0.053 | 0.882 | 0.601 | 0.644 | 0.264 | 0.947 |
|  | 3 | 1.000 | 0.028 | 0.545 | 0.064 | 0.179 | 0.881 | 0.125 | 0.112 | 0.016 | 0.064 | 0.936 | 0.784 | 0.215 | 0.847 | 0.019 | 0.618 | 0.117 | 0.211 | 0.952 | 0.924 | 1.000 | 0.993 | 0.629 | 0.740 |
|  | 10 | 0.006 | 0.256 | 0.013 | 0.001 | 0.026 | 0.025 | 0.001 | 0.006 | 0.003 | 0.100 | 0.750 | 0.988 | 0.306 | 0.014 | 0.027 | 0.109 | 0.995 | 0.951 | 0.391 | 0.543 | 0.881 | 0.098 | 0.001 | 0.047 |
